# Profiling of phenolic composition in camellia oil and its correlative antioxidant properties analysis

**DOI:** 10.3389/fnut.2024.1440279

**Published:** 2024-08-23

**Authors:** Jiarong She, Qingyang Li, Maokai Cui, Qiong Zheng, Jie Yang, Tiantian Chen, Danyu Shen, Shaofeng Peng, Chi Li, Yihua Liu

**Affiliations:** ^1^Hunan Academy of Forestry, Changsha, China; ^2^Research Institute of Subtropical Forestry, Chinese Academy of Forestry, Fuyang, China; ^3^Hunan Shennongguo Oil Eco-Agriculture Development Co., Ltd., Leiyang, China

**Keywords:** camellia oil, phenolic, TPC, antioxidant, OPLS-DA, WGCNA

## Abstract

Less research has been conducted on the association between camellia oil’s (CO) phenolic composition and antioxidant capability. In this study, the phenolic profile of CO and its connection to antioxidant capacity were examined utilizing a combination of widely-targeted phenolic metabolomics and multivariate statistical analysis. A total of 751 phenolics were discovered. The WGCNA was used to link phenols to antioxidants, yielding 161 antioxidant-related phenols from the blue module. In response to several antioxidant assays, 59 (FRAP), 59 (DPPH), and 53 (ABTS) phenolics were identified as differential phenolic markers (DPMs). Further stepwise multiple linear regression revealed six DPMs that substantially influenced the antioxidant capacities. Nine metabolic pathways and their associated network mechanisms for the most significant phenolics were developed. This study sheds light on the phenolic content of CO, elucidates their role in antioxidant activity, and lays the groundwork for improving extraction techniques and generating improved product.

## 1 Introduction

In recent years, the interest in plant-based edible oils with health-promoting properties has surged due to their rich bioactive components (1). Among these, oil extracted from the seeds of Camellia oleifera has garnered significant attention for its unique nutritional profile and potential health benefits. Camellia oil (CO) is also known as ‘eastern olive oil’, due to the similarity in fatty acid profile and physicochemical properties to olive oil. CO has also been listed as a medicinal oil in the Materia Medica and recommended by Food and Agriculture Organization (FAO) as a healthy cooking oil (2, 3). Many studies have shown that the health-promoting properties of CO are mainly due to its balanced fatty acid profile and the antioxidant effect of its phenolic fraction (4, 5). These components with various biological activities are useful for lowering triglycerides (TAGs) and cholesterol, thus preventing hypertension, heart disease, arteriosclerosis, and other diseases (6). Phenolic can also affect oil stability, sensory and nutritional characteristics significantly, and may prevent oil deterioration through quenching radical reactions responsible for lipid oxidation (7). In addition, as naturally-occurring antioxidants, phenolic compounds are more acceptable to consumers than synthetic antioxidants for safety concerns (8). Thus, profiling of phenolic compounds in oil is of great significant for understanding their potential health benefits to human beings and the comprehensive utilization of phenolic compounds.

Composition distribution of phenolic compounds varied in oilseeds. Isoflavones, sinapic acid derivatives, catechin and epicatechin, phenolic alcohols, chlorogenic acid, and lignans were the main phenolic compounds in soybean, rapeseed, peanut skin, olive, sunflower seed, sesame, and flaxseed, respectively (8). The predominant phenolic compounds found in olive oil are oleuropein and its hydrolytic products, hydroxytyrosol, and tyrosol. Hydroxytyrosol has a strong antioxidant effect and the addition of hydroxytyrosol to olive oil can decrease oxidation progress of the oil (9). The link between olive oil consumption, phenolic compounds and health is so solid, that the European Union approved in 2012 a specific health claim on virgin olive oil (VOO) containing at least 5 mg of hydroxytyrosol and derivatives (e.g., oleuropein complex and tyrosol) per 20 g of olive oil (10). Compared to other vegetable oils, less is known about the phenolic composition of CO, especially their relationship with antioxidant activity. Most studies focus on the determination of total phenol content (TPC) in CO (2, 11). A study indicates that CO possesses higher total phenolic content and exhibits stronger *in vitro* antioxidant capacity compared to olive oil and peanut oil. *In vivo*, CO also showed excellent protective effect on S cerevisiae cells, decreased MDA content and ROS level, inhibited CAT, POD and GR enzyme activity (12).

In recent years, several researchers have begun to apply mass spectrometry to explore the phenolic components of CO. Wang et al. analyzed the phenolics of COs from three species gathered from 15 regions of China and identified 24 phenolics (2). However, the assessment of this fraction is quite challenging as the compounds of interest form a rather complex set of analytes, with high chemical diversity and that are found in wide and variable concentration ranges. Metabolomics has recently proven itself to be a powerful tool with which to tackle a broad range of issues related to the analysis of vegetable oil, including its quality, bioactive fraction, sensory features and authenticity (13). A total of 105 phenolics in 22 species were identified by non-targeted metabolomics in CO after three different oil pretreatments (14). In another work for CO, 162 components were tentatively identified, consisting of 76 phenolic acids, 33 flavonols, 22 flavones, 12 flavan-3-ols, 11 flavanones, five stilbenes and three others using ultra-performance-liquid-chromatography tandem quadrupole time-of-flight mass-spectrometry (UPLC Q-TOF MS), in which gallic acid derivatives of phenolic acids, kaempferol derivatives of flavonols, and dimer of flavan-3-ols were the chief phenolic profiles (15). While the previous studies have explored the presence of specific phenolic compounds, there exists a critical gap in understanding the complete phenolic profile at a molecular level and how it relates to CO’s overall antioxidant capacity.

This study aims to address this lacuna by providing an in-depth analysis of the phenolic composition within CO and establishing a robust correlation between the identified phenolics and their antioxidant activities. By employing the combination of widely-target phenol metabolomics and multivariate statistical analysis, we intend to systematically characterize and quantify the diverse array of phenolic compounds present in the oil. The significance of this research lies not only in enhancing our fundamental understanding of the chemical complexity of CO but also in potentially uncovering novel bioactive compounds that could be exploited for their therapeutic potential. Furthermore, a clear elucidation of the relationship between the phenolic composition and antioxidant properties would substantiate the oil’s market positioning as a premium health-enhancing food product and guide future strategies for improving extraction processes and product development.

## 2 Materials and methods

### 2.1 Chemicals and reagents

HPLC-grade methanol (MeOH) and acetonitrile (ACN) were procured from Merck in Hangzhou, China. Formic acid (FA) was sourced from Merck in Aladdin, China. All other solvents, of analytical grade, were obtained from Shanghai GuoYao Chemical Reagents (Shanghai, China).

### 2.2 Preparation of camellia oil samples

The camellia oilseeds used in this study were from Hunan Province and the varieties belonged to Xianglin series. Approximately 20 kg of camellia fruits, all exhibiting the same degree of cracking to ensure uniform maturity, were randomly collected from the site. The hulls were manually removed, and the seeds were subsequently dried using hot air at 60°C until their moisture content ranged between 6 and 8%. The CO was then extracted from the seeds via screw pressing using a ZYJ-420 screw oil press (Hubei Yijiaoyi Machinery Equipment Group Co., Ltd., Hubei, China). The oil content of all camellia oilseeds was greater than 30%. The CO was centrifuged after natural precipitation for more than 20 h under light-avoidance conditions. The upper oil layer from each sample was separated and refrigerated before undergoing further extraction.

### 2.3 Extraction of phenolic compounds

The CO (5 g) was extracted with 50 mL of 80% Methanol–water solution in a cold-water bath. The extraction process was repeated three times, and then the combined liquid extracts were photoevaporated to remove the methanol. After the pH of the liquid extracts was adjusted using 2 M HCl to 2.0, liquid–liquid extraction was carried out using ethyl acetate (three times). The supernatants were evaporated and then re-dissolved in MS-grade methanol to obtain the phenolics.

### 2.4 Phenol metabolome analysis

#### 2.4.1 Sample preparation and extraction

The frozen samples were carefully retrieved from an ultra-low temperature freezer (−80°C). The samples were then subjected to a controlled thawing process under ambient conditions. Once fully thawed, each sample was thoroughly mixed using vortex agitation for 30 s to ensure complete homogeneity. A precise volume of 500 μL was aliquoted from each sample into a clean microcentrifuge tube. Subsequently, 1,000 μL of a 70% methanol solution, fortified with an appropriate internal standard to ensure quantitative accuracy, was added to each tube. The mixture was vortexed for 3 min to promote complete solvation and extraction of the analytes of interest. Thereafter, the tubes were sealed and incubated at 4°C in a refrigerated storage unit overnight. After the incubation period, the mixtures were re-agitated by vertexing for an additional 3 min, followed by a brief ultrasonication for 30 s to eliminate any air bubbles or foam that could potentially interfere with subsequent processing steps. The samples were then centrifuged at 12,000 rpm at 4°C for 3 min to achieve phase separation and clarify the supernatant. Subsequently, 800 μL of the organic layer was carefully collected. The collected solution was filtered through a 0.22 μm microporous membrane and stored in an injection vial for UPLC-MS/MS analysis.

#### 2.4.2 UPLC conditions

The sample extracts were analyzed using a UPLC-ESI-MS/MS system (ExionLC™ AD, AB Sciex, Singapore) coupled with tandem mass spectrometry. The analytical conditions were as follows: UPLC Column: Agilent SB-C18 (1.8 μm, 2.1 mm × 100 mm). Mobile Phase: Solvent A: Pure water with 0.1% formic acid; Solvent B: Acetonitrile with 0.1% formic acid. Gradient Program: 0–9 min: A: B (95%: 5–5%: 95%), 9–10 min: A: B (5%: 95–5%: 95%), 10–11.1 min (5%: 95–95%: 5%), 11.1–14 min (95%: 5–95%: 5%). Flow Rate: 0.35 mL/min. Column Temperature: 40°C. Injection Volume: 2 μL.

#### 2.4.3 ESI-Q trap-MS/MS

The ESI source operation parameters were as follows: The source temperature was set to 550°C. The ion spray voltage (IS) was set to 5,500 V in positive ion mode and −4,500 V in negative ion mode. The ion source gas I (GSI), gas II (GSII), and curtain gas (CUR) were set at 50, 60, and 25 psi, respectively. The collision-activated dissociation (CAD) was set to high. QQQ scans were acquired as MRM (Multiple Reaction Monitoring) experiments, and the collision gas (nitrogen) was set to medium. The DP (declustering potential) and CE (collision energy) for each MRM transition were optimized. A specific set of MRM transitions was monitored for each period based on the eluted metabolites during that period. The mass spectrometry data were processed using the software Analyst 1.6.3. The multi-peak plots for MRM metabolite detection can be seen in Supplementary Figure S1.

#### 2.4.4 Quality control

QC samples are prepared from a mixture of sample extracts and are used to analyze the reproducibility of the samples under the same treatment method. During instrumental analysis, one QC sample is inserted into every 10 samples to monitor the reproducibility of the analytical process, and the reproducibility of metabolite extraction and detection is determined by overlaying and displaying the total ion flow diagrams of the mass spectrometry analyses of different QC samples. Meanwhile, the high stability of the instrument provides an important guarantee for the reproducibility and reliability of the data. The total ion flow plots of QC samples and the overlaid total ion flow plots of different QC samples analyzed by mass spectrometry detection are shown in Supplementary Figures S2, S3, respectively.

### 2.5 Determination of antioxidant capacity

#### 2.5.1 FRAP assay

The FRAP (Ferric Reducing Antioxidant Power) method was conducted following the experimental procedure described in the report by Wang et al. (16). For the assay, 20 μL of camellia oil phenol extract, appropriately diluted, was added to a 96-well plate. Then, 180 μL of FRAP working solution was added to each well. The reaction mixture was incubated for 5 min at 37°C in an oven, protected from light. Subsequently, the absorbance was measured at a wavelength of 593 nm using a microplate reader (Multiskan FC, ThermoFisher, China). The antioxidant capacity of the samples was expressed as Vitamin C (VC) equivalents. The linear regression equation of vitamin C standard calibration curve is shown in Supplementary Figure S4A.

#### 2.5.2 DPPH assay

The radical scavenging activities of the DPPH (1,1-diphenyl-2-picrylhydrazyl) assay were measured following the protocol described by Hazli et al. (17). Briefly, 1 mL of the sample solution with varying concentrations was mixed separately with 4 mL of DPPH solution (10^−4^ mol/L). The mixture was allowed to react for 30 min at a temperature of 37°C. After the reaction, the absorbance values were measured at 517 nm using a microplate reader (Multiskan FC, ThermoFisher, China). The antioxidant activity was quantified by measuring DPPH clearance and expressed as VC equivalent. The linear regression equation of vitamin C standard calibration curve is shown in Supplementary Figure S4B.

#### 2.5.3 ABTS assay

The ABTS [2,2′-azino-bis(3-ethylbenzothiazoline-6-sulfonic acid)] method used in this study was adopted from the report by Wu et al. (18). The working solution of ABTS was prepared by diluting it with water until the absorbance at 734 nm reached 0.63. To measure the antioxidant activity, 200 μL of oil phenol extract, appropriately diluted, was added to a 2 mL centrifuge tube. Then, 1 mL of ABTS working solution was added to the tube, and the mixture was allowed to react at 37°C for 5 min. Subsequently, the absorbance at 734 nm was measured using a microplate reader (Multiskan FC, ThermoFisher, China). The antioxidant activity of the samples was expressed as VC equivalents. The linear regression equation of vitamin C standard calibration curve is shown in Supplementary Figure S4C.

### 2.6 Determination of TPC

The total phenolic content (TPC) was determined using the Folin–Ciocalteu method (19). Briefly, 1 mL of phenol extracts was pipetted into a colorimetric tube. Then, 5 mL of water, 1 mL of Folin reagent, and 3 mL of 75 g/L Na_2_CO_3_ were added sequentially. The reaction solution was allowed to stand for 2 h at room temperature, protected from light. Afterward, the absorbance value was measured at 765 nm using a spectrophotometer (Perkin Elmer, USA).

### 2.7 Data analysis

Statistical significance of the data and linear regression modeling were determined by SPSS 22.0. A metabolite co-expression network was constructed utilizing the weighted gene co-expression network analysis (WGCNA) R package (v 4.2.2). To visualize group segregation and identify significantly altered metabolites, Orthogonal Partial Least-Squares Discriminant Analysis (OPLS-DA) was applied, with differential metabolic markers identified based on VIP > 1. The identified metabolites were annotated using the Kyoto Encyclopedia of Genes and Genomes (KEGG) Compound Database and subsequently mapped to the KEGG Pathway Database. Data visualization was carried out using Metware Cloud^1^ and Origin2019.

## 3 Results

### 3.1 Phenolic composition overview

**Figure 1:**
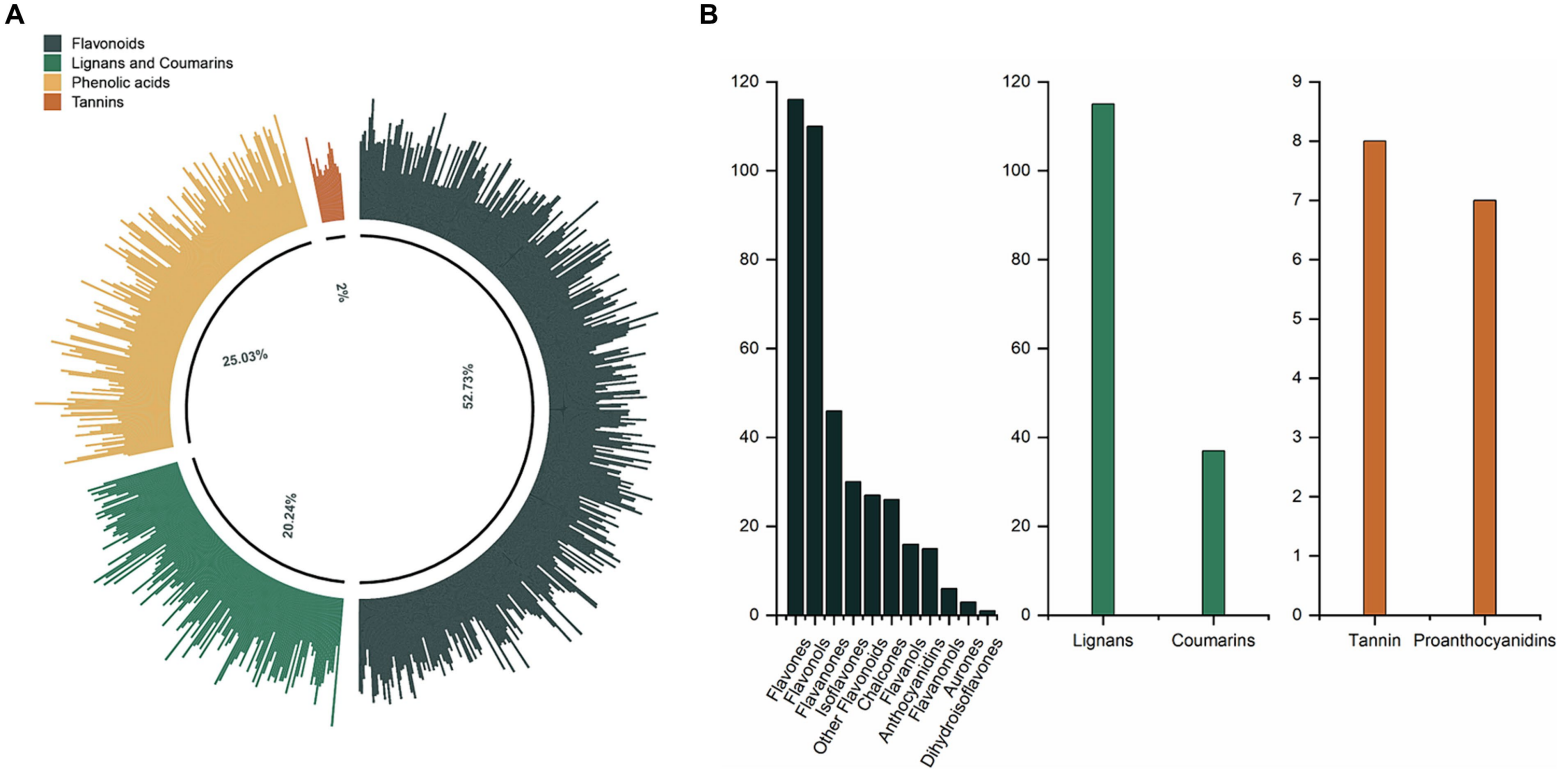
**(A)** The phenolic profile of CO. **(B)** The composition and number of phenolic subclasses in CO.

A total of 751 phenolic compounds were tentatively identified in the oils under analysis (Supplementary Table S1). These phenolics were systematically classified into four categories and 16 subclasses (Figures 1A,B). Flavonoids comprised the predominant major categories, accounting for 52.73% of the total phenolics. They consisted of 116 flavones, 110 flavonols, 46 flavanones, 30 isoflavones, 26 chalcones, 16 flavanols, 15 anthocyanidins, six flavanonols, three aurones, one dihydroisoflavones and 27 other flavonoids. Phenolic acids were the second largest category, accounting for 25.03% of all phenolics. Following that, the Lignans and Coumarins category had 115 lignans and 37 coumarins, accounting for 20.24% of total phenolics. Tannins were the least prevalent category, with just 15 species (eight tannins and seven proanthocyanidins).

### 3.2 TPC and antioxidant capacity

**Figure 2:**
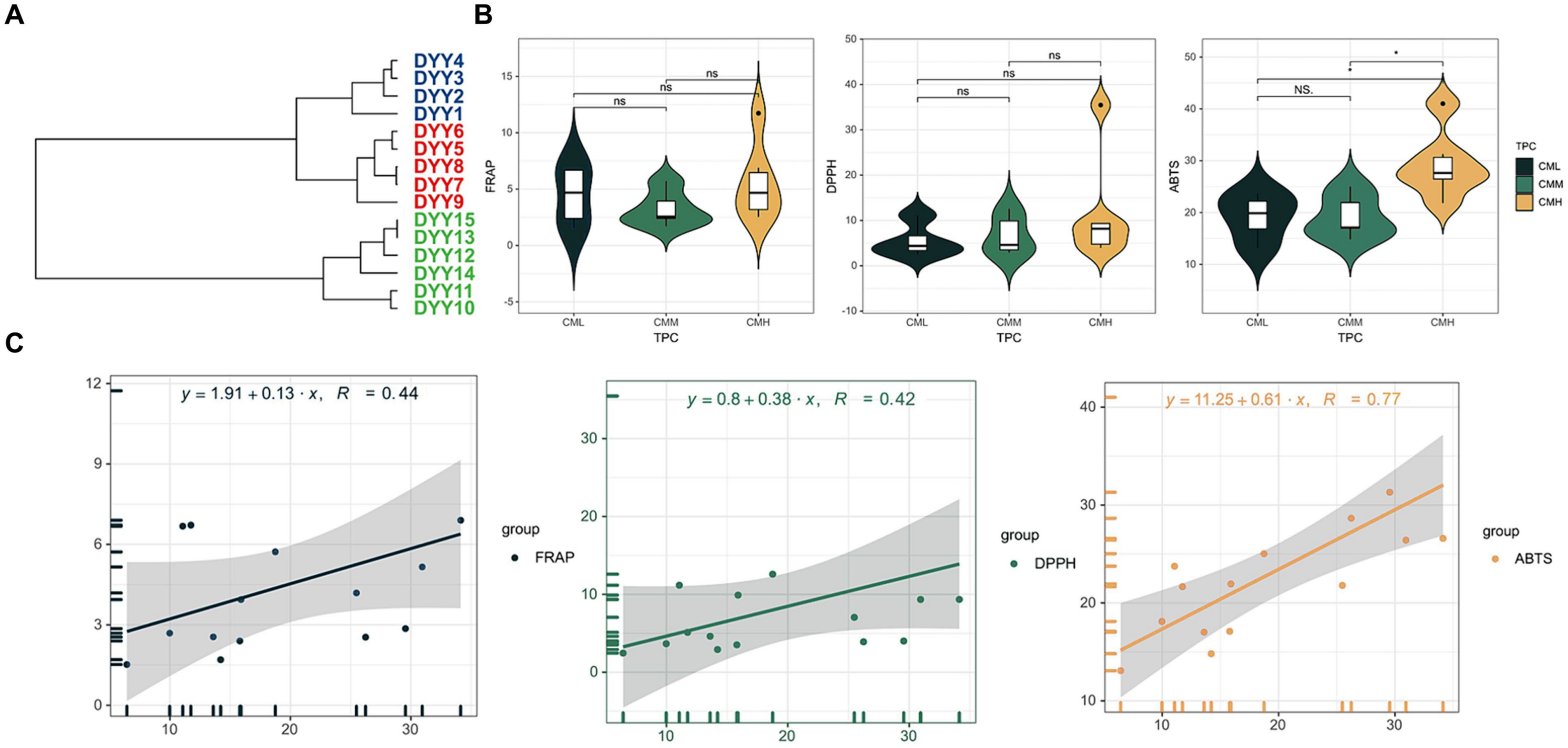
**(A)** Hierarchical clustering of 15 CO samples based on TPC results. **(B)** The violin plots of the three antioxidant results in the high TPC and low TPC groups. **(C)** The linear regression equations of TPC with the three antioxidant results.

The TPC of the analyzed oils varied, with values ranging from 6.43 mg/g for DYY1 to 34.14 mg/g for DYY14. The oils were categorized into three distinct groups based on their TPC levels: the low TPC group (CML), the medium TPC group (CMM), and the high TPC group (CMH). This classification was achieved through hierarchical clustering using the Ward’s D2 method, yielding average TPC values of 9.80 mg/g, 15.65 mg/g, and 29.55 mg/g for each group, respectively (Figure 2A). A one-way ANOVA analysis was conducted to compare the antioxidant capacities of the oils across the different TPC groups, as assessed by three distinct antioxidant assays (Figure 2B and Supplementary Table S2). The analysis revealed significant differences in the ABTS assay, with the CMH group showing the highest ABTS value of 29.29 μg VC/mL, which was more than 30% greater than the values observed for the other two groups.

To investigate the correlation between TPC and antioxidant capacity in COs, a linear regression model was employed (Figure 2C). The results indicated a robust positive correlation between TPC and the antioxidant capacity as measured by the ABTS assay, with a correlation coefficient of 0.77. In contrast, the correlation coefficients for the FRAP and DPPH assays were below 0.5, suggesting a weaker association between TPC and the antioxidant capacities determined by these assays.

### 3.3 WGCNA results

**Figure 3:**
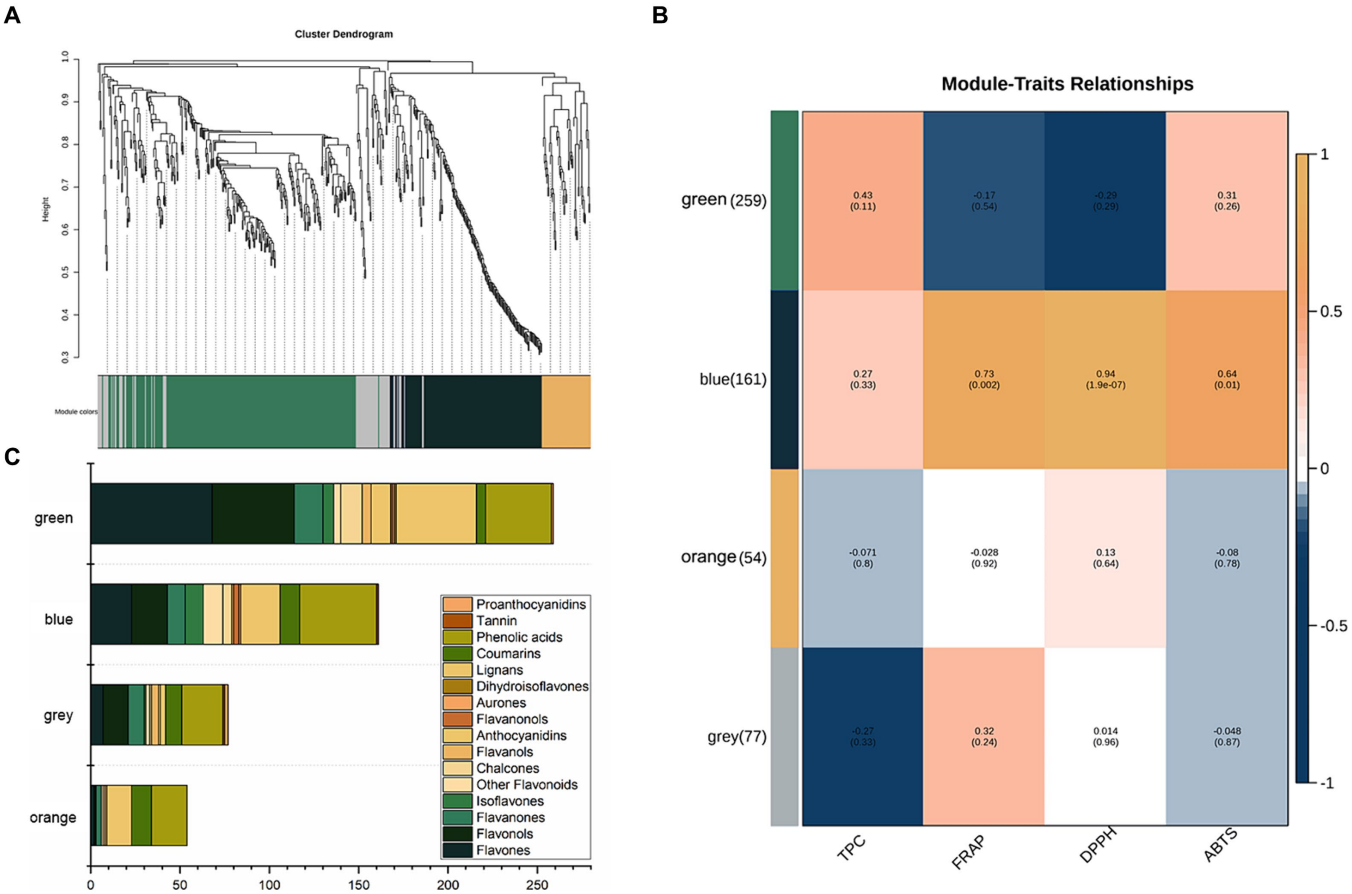
**(A)** Clustering dendrogram of the average network adjacency for the identification of metabolite co-expression modules. **(B)** Module-trait relationships. **(C)** Distribution of different types of metabolites in the four modules.

A co-expression network was built using WGCNA (Weighted Gene Co-expression Network Analysis) to explore the interrelationships among 751 phenolic compounds, TPC, and the results of three antioxidant assays. The co-expression network identified four co-expression modules, which were subsequently grouped into two primary branches characterized by contrasting correlation patterns (Figure 3A). Figure 3B shows the correlation coefficients between the modules and the phenolics. The blue module exhibited significant positive correlations with all three antioxidant assays (FRAP: *r* = 0.73, *p* < 0.05; DPPH: *r* = 0.94, *p* < 0.05; ABTS: *r* = 0.64, *p* < 0.05). However, this module had a weak correlation with TPC, with a correlation coefficient less than 0.30. The blue module included 23 flavones, 20 flavonols, 10 flavanones, 10 isoflavones, five chalcones, three flavanonols, one aurone, one flavanol, 22 lignans, 11 coumarins, 43 phenolic acids, one tannin, and 11 additional flavonoids (Figure 3C).

### 3.4 OPLS-DA analysis

**Figure 4:**
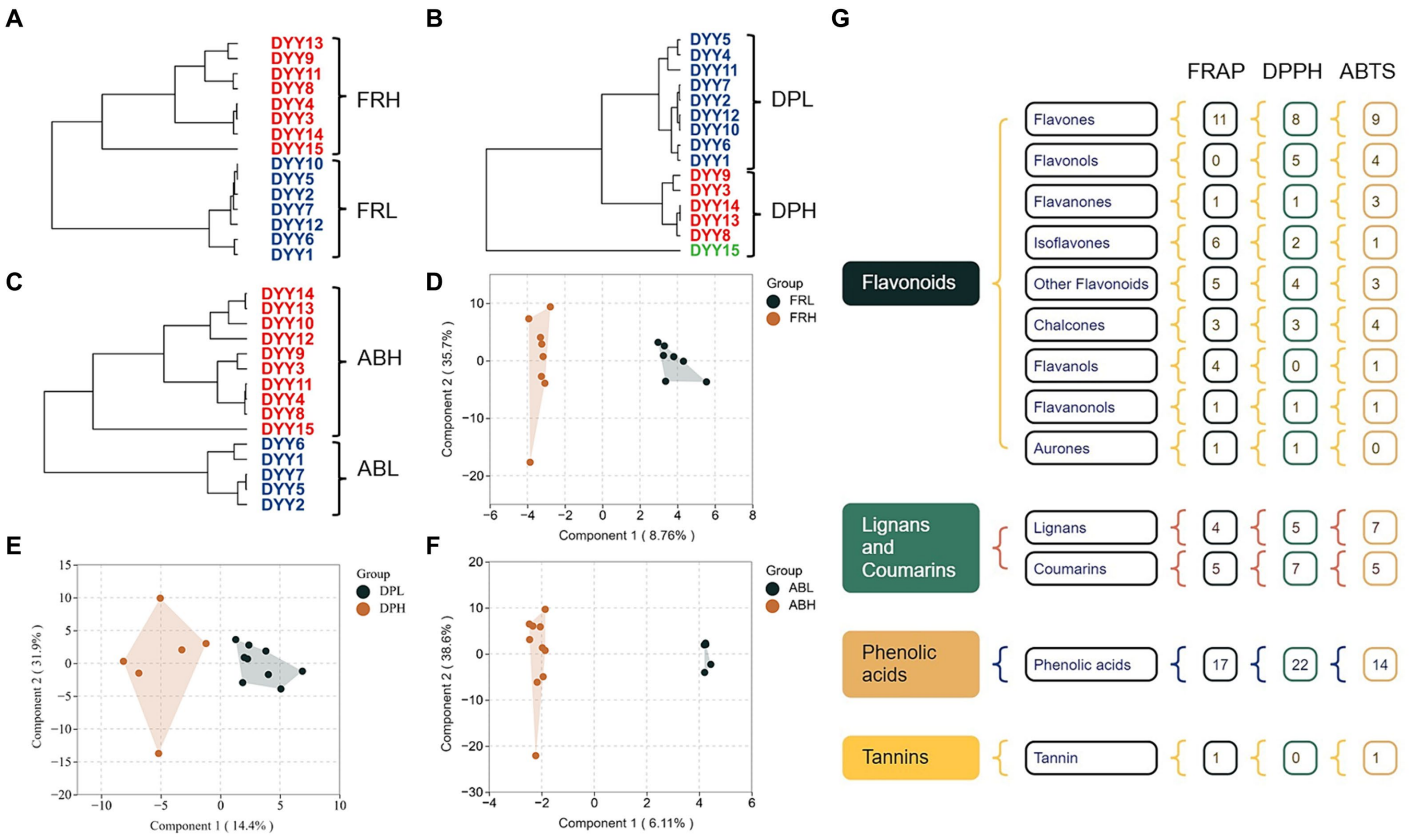
**(A)** Hierarchical clustering of 15 CO samples based on FRAP results. **(B)** Hierarchical clustering of 15 CO samples based on DPPH results. **(C)** Hierarchical clustering of 15 CO samples based on ABTS results. **(D)** OPLS-DA plot of phenolics between COs under FRL and FRH group. **(E)** OPLS-DA plot of phenolics between COs under DPL and DPH group. **(F)** OPLS-DA plot of phenolics between COs under FRL and FRH group. **(G)** Monomer phenol classifications with VIP > 1 based on OPLS-DA results.

The three antioxidant methods were categorized into separate groups using hierarchical clustering: FRL vs. FRH (Figure 4A), DPL vs. DPH (Figure 4B), and ABL vs. ABH (Figure 4C), based on their respective assay findings. To identify the key phenolics influencing the determination of different antioxidant capacities in COs, an OPLS-DA was performed on 161 phenolics from the blue module, which were selected from both the low and high antioxidant activity groups, utilizing data derived from WGCNA. The resulting score plots (Figures 4D–F) demonstrate a pronounced separation between the high and low antioxidant activity groups across all three antioxidant assays. Moreover, examination of the 161 phenolic variables revealed that a subset of 59, 59, and 53 differential phenolic markers (DPM) contributed significantly to the differentiation of the FR, DP, and AB groups, respectively (Figure 4G). Notably, the monophenols, skullcapflavone II, 2,4,4′-trihydroxychalcone, and 3,3′,4-O-trimethylellagic acid emerged as the most influential compounds in the separation of the FR, DP, and AB groups. These compounds had VIP values of 2.34, 2.27, and 2.90, respectively, indicating their substantial impact on the intergroup distinctions observed in the antioxidant assays.

### 3.5 DPMs analysis

**Figure 5:**
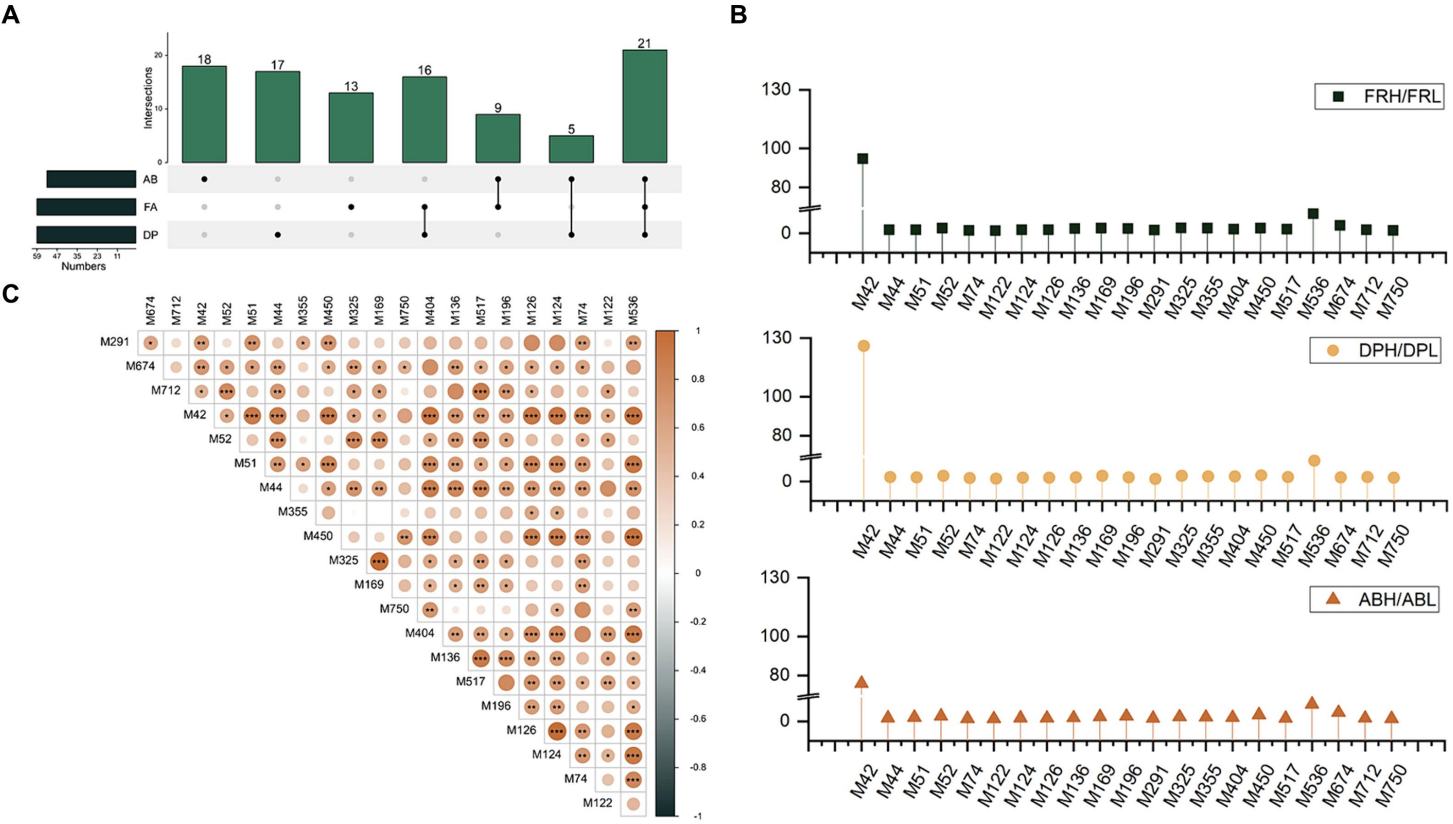
**(A)** The Venn-UpSet diagram of DPMs among three antioxidant assays groups. **(B)** Ratio of the content of 21 co-differential phenols in the high antioxidant group to the low antioxidant group under three antioxidant assays. **(C)** The correlation between the 21 co-differentiated phenols.

As depicted in the Venn-UpSet diagram (Figure 5A), the comparative groups of antioxidant assays share 21 common differential phenolic markers (DPMs), which are predominantly composed of eight phenolic acids, four coumarins, three chalcones, and three flavones (Supplementary Table S3). Among these 21 DPMs, the range of multiplicity of difference was observed to be from 1.37 to 94.68 for FRL vs. FRH, 1.31–125.91 for DPL vs. DPH, and 1.30–75.95 for ABL vs. ABH (Figure 5B). Two monophenols, 2′,3′,4′,5,7-pentahydroxyflavone (with an average multiplicity of difference of 98.85) and methyl orsellinate (with an average multiplicity of difference of 9.78), are particularly noteworthy for exhibiting the highest multiplicity of difference among the groups, underscoring their substantial influence on antioxidant capacity. Furthermore, Pearson correlation analysis revealed positive correlations among all 21 DPMs (Figure 5C). For instance, the correlation coefficients of skullcapflavone II and the other 20 DPMs varied from 0.27 to 0.76. Additionally, 2′,4,4′-trihydroxychalcone and hydroxy coumestrol exhibited strong positive correlations with most of the other DPMs, with most correlation coefficients exceeding 0.7. Notably, the highest correlation coefficient of 0.99 was observed between 3-methylsalicylic acid and 3-methoxybenzoic acid.

## 4 Discussion

The antioxidant properties of CO have been extensively investigated, with polyphenols recognized as natural antioxidants offering a range of health benefits. Previous literature suggests that the content, composition, and kinds of polyphenols present in CO can be influenced by various natural conditions (2). Additionally, polyphenol extraction conditions (20), analytical methods used for identification (4), and anthropogenic factors such as CO production process (21), and processing temperatures (15) can all have an impact the polyphenol profile. Despite this, there has been limited research on the characterization of phenolic compounds in CO, with most studies focusing on only a few compounds.

In this work, a comprehensive analysis using a widely targeted LC–MS/MS method discovered 751 phenolic compounds in CO. These compounds fell into several categories, including 396 flavonoids, 188 phenolic acids, 152 lignans and coumarins, and 15 tannins. This discovery surpasses the number of compounds reported in earlier research by Wei et al. (15) (162 species), Lu et al. (20) (17 species), and Wang et al. (14) (105 species) (details in Supplementary Table S4). This discrepancy is attributed not only to the larger quantities of the same chemicals found in this study but also to the identification of previously unknown subclasses such as aurones and proanthocyanidins.

Previously studies have established a direct link between TPC and antioxidant capacity in fruits (22, 23). Our earlier research found a strong correlation (0.82–0.92) between antioxidant activity and TPC in walnuts (18). However, investigations on plant oils have yielded inconsistent results. A study on palm oil reported a strong positive correlation (*r* = 0.66–0.98) between antioxidant capacity assays, (FRAP and DPPH) and TPC (24). Conversely, another study found that extra virgin olive oil and avocado oil, despite having high TPCs, exhibited lower antioxidant characteristics compared to other oils like olive pomace oil and flaxseed oil (25). In this study, the greatest correlation coefficient between the antioxidant capacity determined by the ABTS method and TPC was 0.77 in CO, whereas correlations with DPPH and FRAP methods were below 0.4. These findings are significantly lower than the correlations observed in olive oil (0.91 for ABTS and 0.74 for DPPH) (26). Moreover, like with walnut oil, there may be variations within the testing techniques themselves. TPC in walnut oil showed a significant correlation with DPPH (*r* = 0.86, *p* < 0.01), but not with the other two methods. Additionally, the correlation between TPC and ABTS was just 0.11 (26). This suggests that TPC alone may not be a reliable indicator of plant oils’ antioxidant capacity.

An increasing number of research demonstrate that the health benefits of vegetable oils, notably their antioxidant capacity, are due to bioactive substances (27, 28). Oleuropein and hydroxytyrosol are the primary bioactive components in olive oil that have antioxidant capabilities (10, 29). In contrast, flaxseed oil contains lignans, which are thought to be the key contributors to its antioxidant capacity (10). In this study, a new Antioxidant Response Metabolites (ARM) library, consisting of 99 responsive phenolics (52 flavonoids, 28 phenolic acids, 18 lignans and coumarins and one tannins) identified from previous DPMs analysis, was created to explore their relationship with the antioxidant capacity of CO. A stepwise multiple linear regression analysis was conducted to examine the relationship between monomer phenols (independent variable, X) and antioxidant capacity (dependent variable, Y) in CO. Among the three antioxidant capacity assays, the DPPH model had the highest adjusted *R*^2^ value of 0.974, with the predictive equation for DPPH being DPPH = 1.845 + 1.288 enterodiol + 0.381 wogonin − 0.409 tricetin − 0.216 methyl 3-(3-hydroxy-4-methoxyphenyl) propanoate. The predictive equations for FRAP and ABTS were FRAP = 1.065 + 0.813 3-methylsalicylic acid (*R*^2^ = 0.635) and ABTS = 12.009 + 0.790 3,3′,4-o-trimethylellagic acid (*R*^2^ = 0.595), respectively. Notably, among the six phenolic compounds described, only enterodiol has been associated with plant oil (flaxseed oil) (30, 31). Enterodiol, a metabolite of the main lignan components found in flaxseed oil, exhibits various biological activities, including anticancer and anti-inflammatory properties (30, 31). In a study focusing on the quantification of 25 phenolic targets in COs, chemometric analysis identified several monomeric phenols with a high correlation to the oil’s antioxidant capacity, such as coumaric, ferulic, vanillic, cinnamic, and caprylic acids (16). The use of LC–MS/MS and metabolomics approaches in this study significantly broadens our understanding of the composition and contribution to the antioxidant capacity of oilseeds.

**Figure 6:**
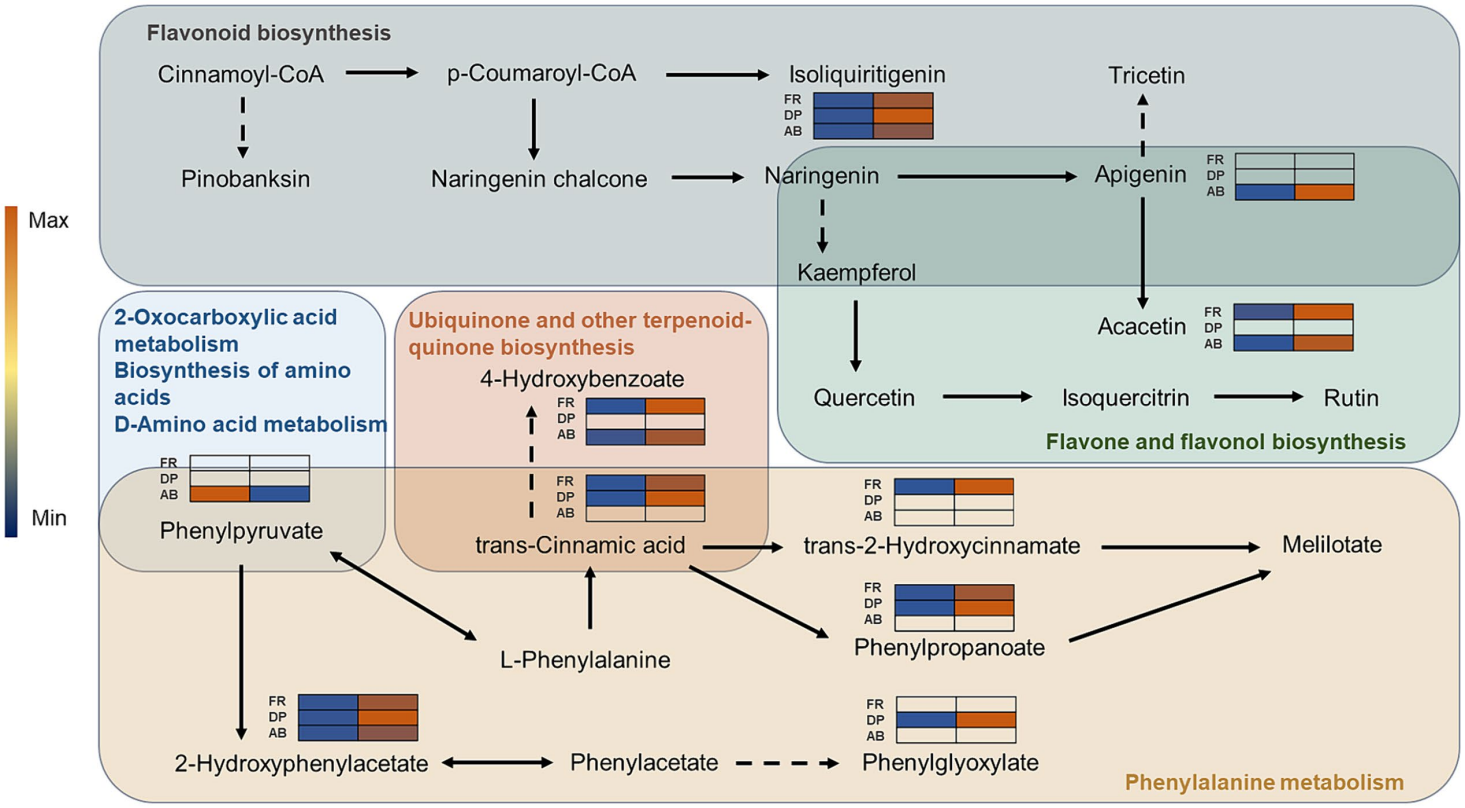
The network of phenolics-related metabolic pathways. The different colored backgrounds represent different metabolic pathways. Solid arrows indicate direct linkages between metabolites and dashed lines indicate indirect linkages. The heatmap shows the relative amount of ARMs in the comparison group of different antioxidant methods.

Following the identification of ARMs by pairwise comparisons, KEGG analysis was utilized to discover all linked metabolic pathways (Supplementary Figure S5). Phenylalanine metabolism and general metabolic pathways were found to be enriched due to the differential metabolism of phenols primarily occurring in the DP and FA groups. Seven metabolic pathways, including flavone and flavonol biosynthesis, were predominantly enriched by differentially metabolized phenols in the AB comparison group. A phenolic pathway network was constructed (Figure 6), revealing apigenin as a key differential phenol in the AB comparison group, acting as a link between two phenolic metabolic pathways with an ABH/ABL ratio of 1.29. Apigenin is converted to tricetin in the flavonoid biosynthesis pathway, influencing the antioxidant outcomes of DPPH assays. It is also converted to tricetin in the flavone and flavonol biosynthesis pathway via methyltransferase, resulting in a differently metabolized phenol in both the FR and AB comparison groups, with relative differences of 1.46 and 1.49, respectively. In the phenylalanine metabolism pathway, L-phenylalanine functions as a precursor to trans-cinnamic acid and interconverts with pinobanksin. Trans-cinnamic acid acts as a differential phenol between the FR and DP groups, with its downstream metabolites, trans-2-hydroxycinnamate and phenylpropanoate, being similarly associated only with the FR and DP groups. Furthermore, trans-cinnamic acid acts as a precursor to 4-hydroxybenzoate in the ubiquinone and other terpenoid-quinone biosynthesis pathway, predominantly affecting FR outcomes. Phenylpyruvate links multiple metabolic pathways and has a significant role in the ABTS comparison results. Two monomer phenols of special interest, 2-hydroxyphenylacetate and isoliquiritigenin, have a considerable effect on the results of all three antioxidant assays.

## 5 Conclusion

For the first time, this study employed a combination of widely-targeted phenolic metabolomics and multivariate statistical analysis to investigate the phenolic profile in CO and its link to antioxidant ability. Using UPLC-MS/MS, a total of 751 phenolics were tentatively identified. The WGCNA results indicated a robust and positive correlation between the blue module, encompassing 161 phenolics, and the outcomes of three distinct antioxidant assays. A detailed examination of these phenolics identified subsets of 59, 59, and 53 differential phenolic markers (DPMs) that played a pivotal role in distinguishing the FR, DP, and AB groups, respectively. Subsequent stepwise multiple linear regression analysis revealed six monomer phenols with significant contribution to antioxidant capacity. Further KEGG enrichment analysis uncovered that nine metabolic pathways, including phenylalanine metabolism, flavone and flavonol, and flavonoid biosynthesis, were significantly enriched. The constructed network of phenolic metabolic pathways elucidated the intricate interplay and relationships between these phenolic compounds at various stages of metabolism. This research significantly advances our comprehension of phenolic compounds in CO, crystallizes the nexus between specific phenolic constituents and their antioxidant capacity, and furnishes valuable insights for future endeavors aimed at refining extraction methodologies and enhancing product development.

## Data availability statement

The original contributions presented in the study are included in the article/Supplementary material, further inquiries can be directed to the corresponding author.

## Author contributions

JS: Writing – original draft, Conceptualization, Visualization, Investigation, Data curation. QL: Writing – original draft, Visualization, Investigation, Formal analysis, Software. MC: Writing – original draft, Visualization, Investigation, Methodology, Formal analysis. QZ: Writing – review & editing, Investigation, Validation, Methodology. JY: Writing – review & editing, Investigation, Validation, Methodology. TC: Writing – review & editing, Investigation, Validation, Methodology. DS: Writing – review & editing, Investigation, Methodology. SP: Writing – review & editing, Investigation. CL: Writing – review & editing, Investigation. YL: Writing – review & editing, Conceptualization, Software, Visualization.

## Footnotes

^1^ https://cloud.metware.cn
