# Supplementary material for: Profiling of phenolic composition in camellia oil and its correlative antioxidant properties analysis
Source: Front Nutr. 2024 Aug 23;11:1440279. doi: 10.3389/fnut.2024.1440279 (PMC11378838; doi:10.3389/fnut.2024.1440279)
Supplement: Supplementary file 1 [file Data_Sheet_1.docx]

Supplementary Material

**Table S1 Information on the classification, molecular weight, ionization model and Q1, Q3 of 751 phenolic compounds**

| Compounds | Class I | Class II | Q1 (Da) | Q3 (Da) | Molecular weight (Da) | Ionization model |
| --- | --- | --- | --- | --- | --- | --- |
| Cyanidin 3-O-sophoroside | Flavonoids | Anthocyanidins | 611.16 | 287.05 | 611.16 | [M]+ |
| Cyanidin-3-O-(6''-O-caffeoyl)glucoside | Flavonoids | Anthocyanidins | 611.14 | 287.06 | 611.14 | [M]+ |
| Cyanidin-3-O-(6''-O-caffeoyl)glucoside-O-malonylglucoside | Flavonoids | Anthocyanidins | 859.2 | 287.05 | 859.20 | [M]+ |
| Cyanidin-3-O-(6''-O-caffeoyl-2''-O-xylosyl)glucoside | Flavonoids | Anthocyanidins | 743.18 | 287.06 | 743.18 | [M]+ |
| Cyanidin-3-O-(6-O-p-coumaroyl-2-O-xylosyl)glucoside | Flavonoids | Anthocyanidins | 727.19 | 287.05 | 727.19 | [M]+ |
| Cyanidin-3-O-[6''-O-(E)-p-coumaroyl-2''-O-xylosyl]glucoside-5-O-glucoside | Flavonoids | Anthocyanidins | 889.24 | 287.05 | 889.24 | [M]+ |
| Cyanidin-3-O-[6''-O-(Z)-p-coumaroyl-2''-O-xylosyl]glucoside-5-O-glucoside | Flavonoids | Anthocyanidins | 889.24 | 287.05 | 889.24 | [M]+ |
| Cyanidin-3-O-glucoside | Flavonoids | Anthocyanidins | 449.11 | 287.06 | 449.11 | [M]+ |
| Cyanidin-3-O-rutinoside (Keracyanin) | Flavonoids | Anthocyanidins | 595.16 | 287.06 | 595.17 | [M]+ |
| Cyanidin-3-O-rutinoside-5,3'-di-O-glucoside | Flavonoids | Anthocyanidins | 919.27 | 449.11 | 919.27 | [M]+ |
| Delphinidin-3-O-rutinoside-7-O-glucoside | Flavonoids | Anthocyanidins | 773.21 | 465.1 | 773.21 | [M]+ |
| Pelargonidin-3-O-rutinoside | Flavonoids | Anthocyanidins | 579.17 | 271.06 | 579.17 | [M]+ |
| Peonidin-3-O-(6''-O-p-coumaroyl)glucoside | Flavonoids | Anthocyanidins | 609.16 | 301.07 | 609.16 | [M]+ |
| Petunidin-3-O-(6''-O-p-coumaroyl)glucoside-5-O-rhamnoside | Flavonoids | Anthocyanidins | 771.21 | 317.07 | 771.21 | [M]+ |
| Petunidin-3-O-(6''-O-p-coumaroyl)rutinoside | Flavonoids | Anthocyanidins | 771.21 | 317.07 | 771.21 | [M]+ |
| Aureusidin | Flavonoids | Aurones | 287.06 | 153.02 | 286.05 | [M+H]+ |
| Aureusidin-4-O-glucoside | Flavonoids | Aurones | 449.11 | 287.06 | 448.10 | [M+H]+ |
| Maritimetin | Flavonoids | Aurones | 285.04 | 185.06 | 286.05 | [M-H]- |
| 2',4,4'-Trihydroxychalcone | Flavonoids | Chalcones | 257.08 | 137.02 | 256.07 | [M+H]+ |
| 2',6'-dimethoxy-4,4'-di-hydroxychalcone | Flavonoids | Chalcones | 301.11 | 181.05 | 300.10 | [M+H]+ |
| 2,4'-Dihydroxy-4,6-dimethoxydihydrochalcone | Flavonoids | Chalcones | 303.12 | 165.06 | 302.12 | [M+H]+ |
| 2,4,2',4'-tetrahydroxy-3'-prenylchalcone | Flavonoids | Chalcones | 341.14 | 137.06 | 340.13 | [M+H]+ |
| 2,4,4'-trihydroxychalcone | Flavonoids | Chalcones | 257.08 | 137.02 | 256.07 | [M+H]+ |
| 3,4,2',4',6'-Pentahydroxychalcone | Flavonoids | Chalcones | 287.06 | 135.05 | 288.06 | [M-H]- |
| 3,4,2',4',6'-Pentahydroxychalcone-4'-O-glucoside | Flavonoids | Chalcones | 449.11 | 287.06 | 450.12 | [M-H]- |
| 3-Prenyl-4,2',4'-Trihydroxychalcone;(licoagrochalcone A) | Flavonoids | Chalcones | 325.14 | 175.07 | 324.14 | [M+H]+ |
| 4'-Hydroxy-3,5-dimethoxydihydrochalcone | Flavonoids | Chalcones | 287.14 | 121.03 | 286.12 | [M+H]+ |
| 4,4'-dihydroxy-2,6-dimethoxydihydrochalcone | Flavonoids | Chalcones | 303.12 | 167.07 | 302.12 | [M+H]+ |
| Carthamone | Flavonoids | Chalcones | 447.09 | 285.04 | 448.10 | [M-H]- |
| Dihydrocharcone-4'-O-glucoside | Flavonoids | Chalcones | 435.13 | 273.08 | 436.14 | [M-H]- |
| Hydroxy isoliquiritigenin glucoside | Flavonoids | Chalcones | 433.11 | 271.06 | 434.12 | [M-H]- |
| Isoliquiritin | Flavonoids | Chalcones | 419.13 | 257.09 | 418.13 | [M+H]+ |
| Isoliquiritin apioside | Flavonoids | Chalcones | 551.18 | 419.13 | 550.17 | [M+H]+ |
| Isosalipurposide (Phlorizin Chalcone) | Flavonoids | Chalcones | 435.13 | 273.09 | 434.12 | [M+H]+ |
| Naringenin chalcone; 2',4,4',6'-Tetrahydroxychalcone | Flavonoids | Chalcones | 273.08 | 153.02 | 272.07 | [M+H]+ |
| Neosakuranin | Flavonoids | Chalcones | 447.13 | 285.08 | 448.14 | [M-H]- |
| Nothofagin | Flavonoids | Chalcones | 437.15 | 107.05 | 436.14 | [M+H]+ |
| Phloretin | Flavonoids | Chalcones | 273.08 | 167.03 | 274.08 | [M-H]- |
| Phloretin-2'-O-(6''-O-rhamnoside)glucoside | Flavonoids | Chalcones | 581.19 | 273.08 | 582.19 | [M-H]- |
| Phloretin-2'-O-(6''-O-xylosyl)glucoside | Flavonoids | Chalcones | 567.17 | 273.08 | 568.18 | [M-H]- |
| Phloretin-2'-O-glucoside (Phlorizin) | Flavonoids | Chalcones | 435.13 | 167.04 | 436.14 | [M-H]- |
| Phloretin-4'-O-glucoside (Trilobatin) | Flavonoids | Chalcones | 435.13 | 273.08 | 436.14 | [M-H]- |
| Pinostrobin Chalcone | Flavonoids | Chalcones | 271.1 | 167.03 | 270.09 | [M+H]+ |
| Sappanchalcone | Flavonoids | Chalcones | 285.08 | 163.04 | 286.08 | [M-H]- |
| 3,4-Dihydro-4-(4'-hydroxyphenyl)-5,7-dihydroxycoumarin glucoside | Lignans and Coumarins | Coumarins | 435.13 | 273.08 | 434.12 | [M+H]+ |
| 3,4-Dihydrocoumarin | Lignans and Coumarins | Coumarins | 147.05 | 119.05 | 148.05 | [M-H]- |
| 3-Methyl-4,8-dihydroxy-3,4-dihydroisocoumarin | Lignans and Coumarins | Coumarins | 193.07 | 101.02 | 194.06 | [M-H]- |
| 4',5'-Dihydropsoralen | Lignans and Coumarins | Coumarins | 189.06 | 131.05 | 188.05 | [M+H]+ |
| 4-Hydroxycoumarin | Lignans and Coumarins | Coumarins | 161.02 | 117.03 | 162.03 | [M-H]- |
| 5,6,7-Trimethoxycoumarin | Lignans and Coumarins | Coumarins | 237.07 | 176.04 | 236.07 | [M+H]+ |
| 5,7,8-trimethoxycoumarin | Lignans and Coumarins | Coumarins | 237.08 | 207.04 | 236.07 | [M+H]+ |
| 5,7-Dimethoxycoumarin (Limettin)(Citropten) | Lignans and Coumarins | Coumarins | 207.07 | 192.04 | 206.06 | [M+H]+ |
| 5,7-dimethoxy-8-hydroxycoumarin | Lignans and Coumarins | Coumarins | 223.06 | 190.03 | 222.05 | [M+H]+ |
| 5,8-Dihydroxypsoralen | Lignans and Coumarins | Coumarins | 219.03 | 173.02 | 218.02 | [M+H]+ |
| 6',7'-Dihydroxybergamottin | Lignans and Coumarins | Coumarins | 373.17 | 203.04 | 372.16 | [M+H]+ |
| 6-Hydroxy-7-methoxycoumarin | Lignans and Coumarins | Coumarins | 193.05 | 133.03 | 192.04 | [M+H]+ |
| 7,8-Dihydrofurocoumarin | Lignans and Coumarins | Coumarins | 189.05 | 131.05 | 188.05 | [M+H]+ |
| 7,8-Dihydroxy-4-methylcoumarin | Lignans and Coumarins | Coumarins | 193.05 | 147.05 | 192.04 | [M+H]+ |
| 7,8-Dihydroxy-4-phenylcoumarin | Lignans and Coumarins | Coumarins | 255.07 | 181.07 | 254.06 | [M+H]+ |
| 7-Hydroxycoumarin-O-rhamnoside | Lignans and Coumarins | Coumarins | 307.08 | 161.02 | 308.09 | [M-H]- |
| 7-Methoxy-coumarin-8-yl-acetaldehyde | Lignans and Coumarins | Coumarins | 219.06 | 161.06 | 218.06 | [M+H]+ |
| 7-methoxy-8-formylcoumarin | Lignans and Coumarins | Coumarins | 205.05 | 175.04 | 204.04 | [M+H]+ |
| 8-Methoxy-6,7-Methylenedioxycoumarin | Lignans and Coumarins | Coumarins | 221.05 | 135.05 | 220.04 | [M+H]+ |
| Avicennol | Lignans and Coumarins | Coumarins | 343.15 | 163.07 | 342.15 | [M+H]+ |
| Decursinol | Lignans and Coumarins | Coumarins | 247.09 | 229.09 | 246.09 | [M+H]+ |
| Desmethylagrimonolide-6-O-glucoside | Lignans and Coumarins | Coumarins | 461.15 | 299.09 | 462.15 | [M-H]- |
| Dimethylfraxetin; 6,7,8-Trimethoxycoumarin | Lignans and Coumarins | Coumarins | 237.07 | 176.05 | 236.07 | [M+H]+ |
| Dimethyllimettin | Lignans and Coumarins | Coumarins | 235.1 | 175.07 | 234.09 | [M+H]+ |
| Ditartaroyl-hydroxycoumarin | Lignans and Coumarins | Coumarins | 427.05 | 295.05 | 426.04 | [M+H]+ |
| Fraxidin (8-Hydroxy-6,7-dimethoxycoumarin) | Lignans and Coumarins | Coumarins | 223.06 | 208.04 | 222.05 | [M+H]+ |
| Hydroxy coumestrol | Lignans and Coumarins | Coumarins | 285.04 | 257.04 | 284.03 | [M+H]+ |
| Methylpicraquassioside A | Lignans and Coumarins | Coumarins | 413.14 | 136.07 | 412.14 | [M+H]+ |
| Peucedanol | Lignans and Coumarins | Coumarins | 265.11 | 173.06 | 264.10 | [M+H]+ |
| Peucedanol 7-O-glucoside | Lignans and Coumarins | Coumarins | 427.16 | 265.11 | 426.15 | [M+H]+ |
| Scoparone | Lignans and Coumarins | Coumarins | 207.07 | 151.08 | 206.06 | [M+H]+ |
| Scopoletin-7-O-glucoside (Scopolin) | Lignans and Coumarins | Coumarins | 355.1 | 193.05 | 354.10 | [M+H]+ |
| Sideretin (5,7,8-Trihydroxy-6-methoxycoumarin) | Lignans and Coumarins | Coumarins | 223.03 | 149.03 | 224.03 | [M-H]- |
| Thunberginol G 3'-Glucoside | Lignans and Coumarins | Coumarins | 435.13 | 273.08 | 434.12 | [M+H]+ |
| coriandrin | Lignans and Coumarins | Coumarins | 231.07 | 201.02 | 230.06 | [M+H]+ |
| dihydrocoriandrin | Lignans and Coumarins | Coumarins | 233.08 | 159.08 | 232.07 | [M+H]+ |
| ulopterol | Lignans and Coumarins | Coumarins | 279.11 | 207.05 | 278.12 | [M+H]+ |
| 2,6,7,4'-Tetrahydroxyisoflavanone | Flavonoids | Dihydroisoflavones | 289.07 | 243.07 | 288.06 | [M+H]+ |
| 3',4,4',5,7-Pentahydroxyflavan (Luteoforol) | Flavonoids | Flavanols | 291.09 | 139.04 | 290.08 | [M+H]+ |
| 3'-O-Methyl-epicatechin | Flavonoids | Flavanols | 305.1 | 139.04 | 304.09 | [M+H]+ |
| 4'-O-Methyl-6-hydroxygallocatechin 3-O-(glutamine ester) | Flavonoids | Flavanols | 465.15 | 319.0841 | 464.14 | [M+H]+ |
| Apiferol | Flavonoids | Flavanols | 275.09 | 139.04 | 274.08 | [M+H]+ |
| Catechin | Flavonoids | Flavanols | 291.09 | 139.04 | 290.08 | [M+H]+ |
| Cinchonain Ib | Flavonoids | Flavanols | 453.11 | 343.08 | 452.11 | [M+H]+ |
| Epiafzelechin | Flavonoids | Flavanols | 275.09 | 107.05 | 274.08 | [M+H]+ |
| Epicatechin glucoside | Flavonoids | Flavanols | 451.12 | 289.07 | 452.13 | [M-H]- |
| Epicatechin | Flavonoids | Flavanols | 291.0863 | 139.0397 | 290.08 | [M+H]+ |
| Epicatechin-(4β→8)-epigallocatechin | Flavonoids | Flavanols | 595.15 | 409.09 | 594.14 | [M+H]+ |
| Epicatechin-3'-O-β-D-glucopyranoside | Flavonoids | Flavanols | 451.13 | 289.07 | 452.13 | [M-H]- |
| Epicatechin-6-C-β-D-glucopyranoside | Flavonoids | Flavanols | 451.13 | 289.07 | 452.13 | [M-H]- |
| Epigallocatechin-3-O-gallate | Flavonoids | Flavanols | 457.0771 | 169.0194 | 458.08 | [M-H]- |
| Fisetinidol-(4α,6)-gallocatechin | Flavonoids | Flavanols | 579.15 | 127.04 | 578.14 | [M+H]+ |
| Gallocatechin 3-O-gallate | Flavonoids | Flavanols | 457.08 | 169.01 | 458.08 | [M-H]- |
| catechin-4-β-D-galactopyranoside | Flavonoids | Flavanols | 451.12 | 289.07 | 452.13 | [M-H]- |
| 2'-Methoxykurarinone | Flavonoids | Flavanones | 453.23 | 179.04 | 452.22 | [M+H]+ |
| 2-hydroxynaringenin | Flavonoids | Flavanones | 287.06 | 125.03 | 288.06 | [M-H]- |
| 4',6-Dihydroxy-5,7-dimethoxyflavanone | Flavonoids | Flavanones | 317.1 | 197.05 | 316.09 | [M+H]+ |
| 5,4'-Dihydroxy-7,8-dimethoxyflavanone | Flavonoids | Flavanones | 317.1 | 197.04 | 316.09 | [M+H]+ |
| 7-O-Methylnaringenin | Flavonoids | Flavanones | 287.09 | 167.03 | 286.08 | [M+H]+ |
| Artocarpanone | Flavonoids | Flavanones | 301.07 | 151 | 302.08 | [M-H]- |
| Blumeatin | Flavonoids | Flavanones | 303.09 | 167.03 | 302.08 | [M+H]+ |
| Carthamidin | Flavonoids | Flavanones | 287.05 | 153.01 | 288.06 | [M-H]- |
| Choerospondin | Flavonoids | Flavanones | 433.11 | 271.06 | 434.12 | [M-H]- |
| Cirsilineol (4',5-Dihydroxy-3',6,7-trimethoxyflavone) | Flavonoids | Flavanones | 343.08 | 313.04 | 344.09 | [M-H]- |
| Dihydrotricetin | Flavonoids | Flavanones | 303.05 | 151.03 | 304.06 | [M-H]- |
| Eriodictyol (5,7,3',4'-Tetrahydroxyflavanone) | Flavonoids | Flavanones | 287.05 | 135.04 | 288.06 | [M-H]- |
| Eriodictyol apiosyl glucoside | Flavonoids | Flavanones | 583.16 | 289.07 | 582.16 | [M+H]+ |
| Eriodictyol-7-O-Rutinoside (Eriocitrin) | Flavonoids | Flavanones | 595.17 | 287.06 | 596.17 | [M-H]- |
| Flowerine | Flavonoids | Flavanones | 369.17 | 298.12 | 368.16 | [M+H]+ |
| Hesperetin-3'-O-glucoside | Flavonoids | Flavanones | 465.14 | 303.09 | 464.13 | [M+H]+ |
| Hesperetin-5-O-glucoside | Flavonoids | Flavanones | 463.12 | 301.07 | 464.13 | [M-H]- |
| Hesperetin-7-O-glucoside | Flavonoids | Flavanones | 465.14 | 303.09 | 464.13 | [M+H]+ |
| Hesperetin-7-O-rutinoside (Hesperidin) | Flavonoids | Flavanones | 609.18 | 301.09 | 610.19 | [M-H]- |
| Homoeriodictyol 7-O-glucoside | Flavonoids | Flavanones | 465.14 | 303.09 | 464.13 | [M+H]+ |
| Isocoreopsin | Flavonoids | Flavanones | 433.11 | 271.06 | 434.12 | [M-H]- |
| Isohemiphloin | Flavonoids | Flavanones | 433.11 | 313.07 | 434.12 | [M-H]- |
| Isookanin | Flavonoids | Flavanones | 289.07 | 153.02 | 288.06 | [M+H]+ |
| Isosakuranetin (5,7-Dihydroxy-4'-methoxyflavanone) | Flavonoids | Flavanones | 287.09 | 153.02 | 286.08 | [M+H]+ |
| Isosakuranetin 7-Alpha-L-Arabinofuranosyl-(1→6)-Glucoside | Flavonoids | Flavanones | 579.17 | 285.08 | 580.18 | [M-H]- |
| Liquiritigenin-4'-O-Glucoside (Liquiritin) | Flavonoids | Flavanones | 419.13 | 257.08 | 418.13 | [M+H]+ |
| Liquiritigenin-7-O-apioside-4'-O-glucoside | Flavonoids | Flavanones | 551.18 | 419.13 | 550.17 | [M+H]+ |
| Methylhesperidin | Flavonoids | Flavanones | 625.21 | 317.1 | 624.21 | [M+H]+ |
| Naringenin (5,7,4'-Trihydroxyflavanone) | Flavonoids | Flavanones | 271.06 | 151 | 272.07 | [M-H]- |
| Naringenin-4',7-dimethyl ether | Flavonoids | Flavanones | 301.11 | 167.04 | 300.10 | [M+H]+ |
| Naringenin-4'-O-glucoside | Flavonoids | Flavanones | 433.12 | 271.06 | 434.12 | [M-H]- |
| Naringenin-6-C-Glucoside | Flavonoids | Flavanones | 435.13 | 285.08 | 434.12 | [M+H]+ |
| Naringenin-7-O-Neohesperidoside(Naringin) | Flavonoids | Flavanones | 579.17 | 271.06 | 580.18 | [M-H]- |
| Naringenin-7-O-Rutinoside(Narirutin) | Flavonoids | Flavanones | 581.19 | 273.08 | 580.18 | [M+H]+ |
| Naringenin-7-O-Rutinoside-4'-O-glucoside | Flavonoids | Flavanones | 741.23 | 579.18 | 742.23 | [M-H]- |
| Naringenin-7-O-glucoside (Prunin) | Flavonoids | Flavanones | 433.11 | 271.07 | 434.12 | [M-H]- |
| O-MethylNaringenin-8-C-arabinoside | Flavonoids | Flavanones | 419.13 | 383.1 | 418.13 | [M+H]+ |
| Persicoside | Flavonoids | Flavanones | 477.14 | 315.09 | 478.15 | [M-H]- |
| Pinocembrin (Dihydrochrysin) | Flavonoids | Flavanones | 257.08 | 153.02 | 256.07 | [M+H]+ |
| Pinocembrin 5-O-glucoside | Flavonoids | Flavanones | 419.13 | 257.08 | 418.13 | [M+H]+ |
| Pinocembrin-7-O-(2''-O-arabinosyl)glucoside | Flavonoids | Flavanones | 549.16 | 255.06 | 550.17 | [M-H]- |
| Pinocembrin-7-O-glucoside (Pinocembroside) | Flavonoids | Flavanones | 419.13 | 257.08 | 418.13 | [M+H]+ |
| Pinocembrin-7-O-neohesperidoside | Flavonoids | Flavanones | 563.18 | 255.07 | 564.18 | [M-H]- |
| Pinocembrin-7-O-rutinoside | Flavonoids | Flavanones | 563.18 | 255.07 | 564.18 | [M-H]- |
| Pinostrobin | Flavonoids | Flavanones | 271.1 | 167.03 | 270.09 | [M+H]+ |
| Pinostrobin 5-O-Glucoside | Flavonoids | Flavanones | 433.15 | 271.1 | 432.14 | [M+H]+ |
| 3,5,7-Trihydroxyflavanone (Pinobanksin) | Flavonoids | Flavanonols | 271.06 | 151 | 272.07 | [M-H]- |
| 3-Hydroxy-4',5,7-Trimethoxyflavanone | Flavonoids | Flavanonols | 331.12 | 161.06 | 330.11 | [M+H]+ |
| Aromadendrin (Dihydrokaempferol) | Flavonoids | Flavanonols | 287.06 | 259.07 | 288.06 | [M-H]- |
| Aromadendrin-7-O-glucoside | Flavonoids | Flavanonols | 449.11 | 287.06 | 450.12 | [M-H]- |
| Phellamurin | Flavonoids | Flavanonols | 519.19 | 357.13 | 518.18 | [M+H]+ |
| Phellodendroside | Flavonoids | Flavanonols | 519.19 | 357.13 | 518.18 | [M+H]+ |
| 2',3',4',5,7-pentahydroxyflavone | Flavonoids | Flavones | 301.04 | 151 | 302.04 | [M-H]- |
| 3',4',5',5,7-Pentamethoxyflavone | Flavonoids | Flavones | 373.13 | 343.08 | 372.12 | [M+H]+ |
| 3'-O-Methyltricetin-5-O-glucoside | Flavonoids | Flavones | 479.12 | 317.06 | 478.11 | [M+H]+ |
| 3'-O-Methyltricetin-7-O-glucoside | Flavonoids | Flavones | 479.12 | 317.07 | 478.11 | [M+H]+ |
| 4',5,7-Trihydroxy-3',6-dimethoxyflavone (Jaceosidin) | Flavonoids | Flavones | 331.0812 | 316.0577 | 330.07 | [M+H]+ |
| 4'-O-Glucosylvitexin | Flavonoids | Flavones | 595.17 | 433.12 | 594.16 | [M+H]+ |
| 5,7,2'-Trihydroxy-8-methoxyflavone | Flavonoids | Flavones | 299.06 | 284.04 | 300.06 | [M-H]- |
| 5,7,2'-Trihydroxy-8-methoxyflavone; Scutevulin | Flavonoids | Flavones | 301.07 | 286.05 | 300.06 | [M+H]+ |
| 5-Hydroxy-4',6,7,8-tetramethoxyflavone | Flavonoids | Flavones | 359.11 | 344.09 | 358.11 | [M+H]+ |
| 6,7,8-Tetrahydroxy-5-methoxyflavone | Flavonoids | Flavones | 301.07 | 286.05 | 300.06 | [M+H]+ |
| 6-Hydroxyluteolin 5-glucoside | Flavonoids | Flavones | 463.09 | 301.03 | 464.10 | [M-H]- |
| Acacetin | Flavonoids | Flavones | 285.08 | 242.05 | 284.07 | [M+H]+ |
| Acacetin-7-O-galactoside | Flavonoids | Flavones | 447.13 | 285.08 | 446.12 | [M+H]+ |
| Acacetin-7-O-neohesperidoside | Flavonoids | Flavones | 593.19 | 285.08 | 592.18 | [M+H]+ |
| Acacetin-7-O-rutinoside (Linarin) | Flavonoids | Flavones | 593.19 | 285.08 | 592.18 | [M+H]+ |
| Apigenin-4'-O-(2''-O-p-coumaroyl)-β-D-glucopyranoside | Flavonoids | Flavones | 579.15 | 271.06 | 578.14 | [M+H]+ |
| Apigenin-4'-O-glucoside | Flavonoids | Flavones | 433.1129 | 271.0736 | 432.11 | [M+H]+ |
| Apigenin-5-O-glucoside | Flavonoids | Flavones | 433.11 | 271.06 | 432.11 | [M+H]+ |
| Apigenin-6,8-di-C-arabinoside | Flavonoids | Flavones | 535.14 | 499.12 | 534.14 | [M+H]+ |
| Apigenin-6,8-di-C-glucoside (Vicenin-2) | Flavonoids | Flavones | 595.17 | 427.1 | 594.16 | [M+H]+ |
| Apigenin-6-C-(2''-xylosyl)glucoside | Flavonoids | Flavones | 565.16 | 433.11 | 564.15 | [M+H]+ |
| Apigenin-6-C-fucoside | Flavonoids | Flavones | 417.12 | 381.1 | 416.11 | [M+H]+ |
| Apigenin-6-C-glucose-8-C-rhamnoside | Flavonoids | Flavones | 579.17 | 423.1 | 578.16 | [M+H]+ |
| Apigenin-6-C-glucoside (Isovitexin) | Flavonoids | Flavones | 431.1 | 311.05 | 432.11 | [M-H]- |
| Apigenin-6-C-rhamnoside | Flavonoids | Flavones | 417.12 | 381.1 | 416.11 | [M+H]+ |
| Apigenin-7-O-(2''-apiosyl)glucoside (Apiin) | Flavonoids | Flavones | 565.16 | 271.06 | 564.15 | [M+H]+ |
| Apigenin-7-O-(2''-glucosyl)arabinoside | Flavonoids | Flavones | 565.16 | 271.06 | 564.15 | [M+H]+ |
| Apigenin-7-O-(6''-p-Coumaryl)glucoside | Flavonoids | Flavones | 579.15 | 271.06 | 578.14 | [M+H]+ |
| Apigenin-7-O-glucoside(Cosmosiin) | Flavonoids | Flavones | 433.11 | 271.07 | 432.11 | [M+H]+ |
| Apigenin-7-O-glucoside-4'-O-rutinoside | Flavonoids | Flavones | 739.21 | 269.04 | 740.22 | [M-H]- |
| Apigenin-7-O-neohesperidoside (Rhoifolin) | Flavonoids | Flavones | 579.17 | 271.06 | 578.16 | [M+H]+ |
| Apigenin-7-O-rutinoside (Isorhoifolin) | Flavonoids | Flavones | 579.17 | 271.06 | 578.16 | [M+H]+ |
| Apigenin-7-O-rutinoside-4'-O-rhamnoside | Flavonoids | Flavones | 725.2 | 433.11 | 724.22 | [M+H]+ |
| Apigenin-8-C-Glucoside (Vitexin) | Flavonoids | Flavones | 433.11 | 313.07 | 432.11 | [M+H]+ |
| Apigenin; 4',5,7-Trihydroxyflavone | Flavonoids | Flavones | 271.06 | 153.02 | 270.05 | [M+H]+ |
| Chrysin | Flavonoids | Flavones | 255.07 | 153.02 | 254.06 | [M+H]+ |
| Chrysin apiosyl glucoside | Flavonoids | Flavones | 549.16 | 255.07 | 548.15 | [M+H]+ |
| Chrysin-5-O-glucoside (Toringin) | Flavonoids | Flavones | 417.12 | 255.07 | 416.11 | [M+H]+ |
| Chrysin-7-O-glucoside | Flavonoids | Flavones | 417.12 | 255.06 | 416.11 | [M+H]+ |
| Chrysin-8-C-glucoside | Flavonoids | Flavones | 417.12 | 297.08 | 416.11 | [M+H]+ |
| Chrysoeriol-7-O-glucoside | Flavonoids | Flavones | 461.11 | 299.06 | 462.12 | [M-H]- |
| Chrysoeriol; 5,7,4'-Trihydroxy-3'-Methoxyflavone | Flavonoids | Flavones | 299.06 | 284.03 | 300.06 | [M-H]- |
| Clitorin | Flavonoids | Flavones | 741.2239 | 287.06 | 740.22 | [M+H]+ |
| Diosmetin (5,7,3'-Trihydroxy-4'-methoxyflavone) | Flavonoids | Flavones | 301.07 | 286.05 | 300.06 | [M+H]+ |
| Diosmetin-7-O-Neohesperidoside (Neodiosmin) | Flavonoids | Flavones | 609.18 | 301.08 | 608.17 | [M+H]+ |
| Diosmetin-7-O-galactoside | Flavonoids | Flavones | 463.12 | 301.07 | 462.12 | [M+H]+ |
| Diosmetin-7-O-glucoside | Flavonoids | Flavones | 463.12 | 301.07 | 462.12 | [M+H]+ |
| Diosmetin-7-O-rutinoside (Diosmin) | Flavonoids | Flavones | 609.18 | 301.08 | 608.17 | [M+H]+ |
| Diosmetin-8-C-(2''-O-rhamnosyl)glucoside | Flavonoids | Flavones | 609.18 | 463.12 | 608.17 | [M+H]+ |
| Disporopsin | Flavonoids | Flavones | 301.073 | 179.03 | 302.08 | [M-H]- |
| Genkwanin-6-C-(2''-O-apiosyl)glucoside | Flavonoids | Flavones | 579.17 | 447.13 | 578.16 | [M+H]+ |
| Hispidulin (5,7,4'-Trihydroxy-6-methoxyflavone) | Flavonoids | Flavones | 301.07 | 286.05 | 300.06 | [M+H]+ |
| Hispidulin-7-O-glucoside(Homoplantaginin) | Flavonoids | Flavones | 463.13 | 301.07 | 462.12 | [M+H]+ |
| Hispidulin-8-C-(2''-O-glucosyl)glucoside | Flavonoids | Flavones | 625.18 | 463.13 | 624.17 | [M+H]+ |
| Hispidulin-8-C-(2''-O-xylosyl)xyloside | Flavonoids | Flavones | 565.16 | 433.11 | 564.15 | [M+H]+ |
| Hypolaetin | Flavonoids | Flavones | 301.04 | 149.02 | 302.04 | [M-H]- |
| Isoetin (5,7,2',4',5'-Pentahydroxyflavone) | Flavonoids | Flavones | 301.04 | 149.03 | 302.04 | [M-H]- |
| Isoorientin-7-O-(2''-rhamnosyl)glucoside | Flavonoids | Flavones | 757.22 | 431.4 | 756.21 | [M+H]+ |
| Isoorientin-7-O-(6''-feruloyl)arabinoside | Flavonoids | Flavones | 757.2 | 177.1 | 756.19 | [M+H]+ |
| Isoorientin-7-O-glucoside | Flavonoids | Flavones | 611.17 | 449.11 | 610.15 | [M+H]+ |
| Isosaponarin(Isovitexin-4'-O-glucoside) | Flavonoids | Flavones | 595.17 | 313.07 | 594.16 | [M+H]+ |
| Isoschaftoside | Flavonoids | Flavones | 565.16 | 409.09 | 564.15 | [M+H]+ |
| Isoscoparin-5-O-(6'''-p-coumaroyl)-glucopyranoside | Flavonoids | Flavones | 771.21 | 433.11 | 770.21 | [M+H]+ |
| Isoscutellarein | Flavonoids | Flavones | 287.06 | 153.01 | 286.05 | [M+H]+ |
| Isovitexin-2''-O-rhamnoside | Flavonoids | Flavones | 579.17 | 313.07 | 578.16 | [M+H]+ |
| Isovitexin-2''-O-xyloside | Flavonoids | Flavones | 565.15 | 433.11 | 564.15 | [M+H]+ |
| Isovitexin-7-O-glucoside(Saponarin) | Flavonoids | Flavones | 595.16 | 313.07 | 594.16 | [M+H]+ |
| Isovitexin-8-O-xyloside | Flavonoids | Flavones | 565.15 | 313.07 | 564.15 | [M+H]+ |
| Jaceosidin-7-O-Glucoside | Flavonoids | Flavones | 493.1354 | 331.08 | 492.13 | [M+H]+ |
| Ladanetin-6-O-β-D-glucoside | Flavonoids | Flavones | 463.13 | 301.08 | 462.12 | [M+H]+ |
| Ligustroflavone(Nuezhenoside) | Flavonoids | Flavones | 725.23 | 271.07 | 724.22 | [M+H]+ |
| Luteolin 7-rutinoside-4'-O-Rhamnoside | Flavonoids | Flavones | 741.23 | 433.12 | 740.22 | [M+H]+ |
| Luteolin-3'-O-glucoside | Flavonoids | Flavones | 449.11 | 287.06 | 448.10 | [M+H]+ |
| Luteolin-4'-O-glucoside | Flavonoids | Flavones | 449.11 | 287.05 | 448.10 | [M+H]+ |
| Luteolin-6,8-di-C-glucoside | Flavonoids | Flavones | 611.16 | 473.11 | 610.15 | [M+H]+ |
| Luteolin-6-C-arabinoside-7-O-glucoside | Flavonoids | Flavones | 581.15 | 383.07 | 580.14 | [M+H]+ |
| Luteolin-6-C-glucoside-7-O-rhamnoside | Flavonoids | Flavones | 595.17 | 449.11 | 594.16 | [M+H]+ |
| Luteolin-7,3'-di-O-glucoside | Flavonoids | Flavones | 611.16 | 287.06 | 610.15 | [M+H]+ |
| Luteolin-7-O-(6''-malonyl)glucoside | Flavonoids | Flavones | 535.11 | 287.05 | 534.10 | [M+H]+ |
| Luteolin-7-O-(6''-malonyl)glucoside-5-O-arabinoside | Flavonoids | Flavones | 667.15 | 287.06 | 666.14 | [M+H]+ |
| Luteolin-7-O-gentiobioside | Flavonoids | Flavones | 611.16 | 287.05 | 610.15 | [M+H]+ |
| Luteolin-7-O-neohesperidoside (Lonicerin) | Flavonoids | Flavones | 595.17 | 287.06 | 594.16 | [M+H]+ |
| Luteolin-7-O-rutinoside | Flavonoids | Flavones | 595.17 | 287.06 | 594.16 | [M+H]+ |
| Luteolin-8-C-arabinoside | Flavonoids | Flavones | 419.09 | 383.07 | 418.09 | [M+H]+ |
| Mosloflavone; 5-Hydroxy-6,7-Dimethoxyflavone | Flavonoids | Flavones | 299.09 | 284.07 | 298.08 | [M+H]+ |
| Nepetin-7-O-glucoside(Nepitrin) | Flavonoids | Flavones | 479.12 | 317.06 | 478.11 | [M+H]+ |
| Nitensoside B | Flavonoids | Flavones | 625.18 | 317.07 | 624.17 | [M+H]+ |
| Nobiletin (5,6,7,8,3',4'-Hexamethoxyflavone) | Flavonoids | Flavones | 403.14 | 373.1 | 402.13 | [M+H]+ |
| Norartocarpetin | Flavonoids | Flavones | 285.04 | 285.04 | 286.05 | [M-H]- |
| Odoratin rutinoside | Flavonoids | Flavones | 623.2 | 315.08 | 622.19 | [M+H]+ |
| Orientin-2''-O-(6'''-(E)-Caffeoyl)-glucopyranoside | Flavonoids | Flavones | 773.19 | 163.04 | 772.19 | [M+H]+ |
| Orientin-2''-O-(6'''-p-coumaroyl)-glucopyranoside | Flavonoids | Flavones | 757.2 | 449.11 | 756.19 | [M+H]+ |
| Orientin-2''-O-rhamnoside | Flavonoids | Flavones | 595.16 | 449.11 | 594.16 | [M+H]+ |
| Orientin-7-O-glucoside | Flavonoids | Flavones | 611.16 | 449.11 | 610.15 | [M+H]+ |
| Oroxin A | Flavonoids | Flavones | 433.11 | 271.06 | 432.11 | [M+H]+ |
| Pedaliin | Flavonoids | Flavones | 479.12 | 317.08 | 478.11 | [M+H]+ |
| Phloretin 3',5'-Di-C-Glucoside | Flavonoids | Flavones | 599.198 | 431.1341 | 598.19 | [M+H]+ |
| Scutellarein-4'-methyl ether (5,6,7-Trihydroxy-4'-methoxyflavone) | Flavonoids | Flavones | 301.07 | 258.05 | 300.06 | [M+H]+ |
| Selagin | Flavonoids | Flavones | 317.06 | 302.04 | 316.06 | [M+H]+ |
| Skullcapflavone II | Flavonoids | Flavones | 375.11 | 360.08 | 374.10 | [M+H]+ |
| Sorbifolin | Flavonoids | Flavones | 301.07 | 286.05 | 300.06 | [M+H]+ |
| Tangeretin (4',5,6,7,8-Pentamethoxyflavone) | Flavonoids | Flavones | 373.13 | 343.09 | 372.12 | [M+H]+ |
| Tectochrysin | Flavonoids | Flavones | 269.08 | 226.07 | 268.07 | [M+H]+ |
| Tricetin (5,7,3',4',5'-Pentahydroxyflavone) | Flavonoids | Flavones | 301.04 | 151 | 302.04 | [M-H]- |
| Tricin-4'-O-glucoside | Flavonoids | Flavones | 493.13 | 331.08 | 492.13 | [M+H]+ |
| Tricin-7-O-Glucoside | Flavonoids | Flavones | 493.13 | 331.08 | 492.13 | [M+H]+ |
| Tricin-7-O-saccharic acid | Flavonoids | Flavones | 521.09 | 329.2 | 522.10 | [M-H]- |
| Vaccarin | Flavonoids | Flavones | 727.21 | 433.11 | 726.20 | [M+H]+ |
| Vicenin-3 | Flavonoids | Flavones | 563.15 | 353.07 | 564.15 | [M-H]- |
| Violanthin | Flavonoids | Flavones | 579.17 | 525.14 | 578.16 | [M+H]+ |
| Vitexin-2''-O-galactoside | Flavonoids | Flavones | 595.17 | 433.12 | 594.16 | [M+H]+ |
| Vitexin-2''-O-glucoside | Flavonoids | Flavones | 595.16 | 313.07 | 594.16 | [M+H]+ |
| Vitexin-2''-O-rhamnoside | Flavonoids | Flavones | 579.17 | 433.11 | 578.16 | [M+H]+ |
| Vitexin-7-O-(6''-p-coumaroyl)glucoside | Flavonoids | Flavones | 741.2 | 147.3 | 740.20 | [M+H]+ |
| Wogonin (5,7-Dihydroxy-8-Methoxyflavone) | Flavonoids | Flavones | 285.08 | 270.06 | 284.07 | [M+H]+ |
| Yuanhuanin* | Flavonoids | Flavones | 463.12 | 301.08 | 462.12 | [M+H]+ |
| 3'-methoxyquercetin-3-O-L-rhamnosyl(1→2)-glucopyranoside | Flavonoids | Flavonols | 625.17 | 317.07 | 624.17 | [M+H]+ |
| 3,5,3'-Trihydroxy-7,4'-dimethoxyflavone (Ombuin) | Flavonoids | Flavonols | 331.08 | 316.05 | 330.07 | [M+H]+ |
| 3,5,4'-Trihydroxy-7-methoxyflavone (Rhamnocitrin) | Flavonoids | Flavonols | 301.07 | 286.05 | 300.06 | [M+H]+ |
| 3,5,6,7,8,4'-Hexamethoxyflavone | Flavonoids | Flavonols | 403.14 | 373.09 | 402.13 | [M+H]+ |
| 3,5,7,2'-Tetrahydroxyflavone; Datiscetin | Flavonoids | Flavonols | 285.04 | 133.03 | 286.05 | [M-H]- |
| 5,7-dihydroxy-2-(3-hydroxyphenyl)-3-methoxy-4H-chromen-4-one | Flavonoids | Flavonols | 299.06 | 284.03 | 300.06 | [M-H]- |
| 6-C-MethylKaempferol-3-glucoside | Flavonoids | Flavonols | 463.12 | 301.08 | 462.12 | [M+H]+ |
| 6-C-Methylquercetin-3-O-rutinoside | Flavonoids | Flavonols | 625.18 | 317.06 | 624.17 | [M+H]+ |
| 6-Hydroxykaempferol-7-O-glucoside | Flavonoids | Flavonols | 465.1 | 303.1 | 464.10 | [M+H]+ |
| 6-Methoxykaempferol-3-O-glucoside | Flavonoids | Flavonols | 479.12 | 317.07 | 478.11 | [M+H]+ |
| Avicularin(Quercetin-3-O-α-L-arabinofuranoside) | Flavonoids | Flavonols | 435.09 | 303.05 | 434.08 | [M+H]+ |
| Azalein (Azaleatin-3-O-rhamnoside) | Flavonoids | Flavonols | 463.12 | 317.07 | 462.12 | [M+H]+ |
| Biondnoid I | Flavonoids | Flavonols | 595.1446 | 147 | 594.14 | [M+H]+ |
| Fisetin | Flavonoids | Flavonols | 287.06 | 137.02 | 286.05 | [M+H]+ |
| Galangin (3,5,7-Trihydroxyflavone) | Flavonoids | Flavonols | 269.05 | 151 | 270.05 | [M-H]- |
| Galangin 7-glucoside | Flavonoids | Flavonols | 433.11 | 271.06 | 432.11 | [M+H]+ |
| Gossypetin-3-O-rutinoside | Flavonoids | Flavonols | 627.16 | 319.05 | 626.15 | [M+H]+ |
| Gossypetin-3-O-rutinoside-8-O-rhamnoside | Flavonoids | Flavonols | 773.21 | 465.1 | 772.21 | [M+H]+ |
| Gossypetin-8-O-glucoside | Flavonoids | Flavonols | 481.1 | 319.05 | 480.09 | [M+H]+ |
| Isorhamnetin 3-O-a-L-arabinopyranoside 7-O-a-L-rhamnopyranoside | Flavonoids | Flavonols | 595.17 | 317.07 | 594.16 | [M+H]+ |
| Isorhamnetin-3-O-(2''-O-xylosyl)glucoside | Flavonoids | Flavonols | 611.16 | 317.06 | 610.15 | [M+H]+ |
| Isorhamnetin-3-O-(2',6'-di-O-α-L-rhamnopyranosyl)-β-D-glucopyranoside; Typhaneoside | Flavonoids | Flavonols | 771.24 | 317.07 | 770.23 | [M+H]+ |
| Isorhamnetin-3-O-Glucoside | Flavonoids | Flavonols | 479.12 | 317.07 | 478.11 | [M+H]+ |
| Isorhamnetin-3-O-arabinoside | Flavonoids | Flavonols | 449.11 | 317.06 | 448.10 | [M+H]+ |
| Isorhamnetin-3-O-glucoside-7-O-rhamnoside | Flavonoids | Flavonols | 625.18 | 317.07 | 624.17 | [M+H]+ |
| Isorhamnetin-3-O-neohesperidoside | Flavonoids | Flavonols | 625.17 | 317.07 | 624.17 | [M+H]+ |
| Isorhamnetin-3-O-rutinoside (Narcissin) | Flavonoids | Flavonols | 625.18 | 317.08 | 624.17 | [M+H]+ |
| Isorhamnetin-7-O-glucoside (Brassicin) | Flavonoids | Flavonols | 479.12 | 317.06 | 478.11 | [M+H]+ |
| Isoxanthohumol | Flavonoids | Flavonols | 355.15 | 179.04 | 354.15 | [M+H]+ |
| Izalpinin | Flavonoids | Flavonols | 285.08 | 242.06 | 284.07 | [M+H]+ |
| Kaempferol (3,5,7,4'-Tetrahydroxyflavone) | Flavonoids | Flavonols | 287.06 | 153.02 | 286.05 | [M+H]+ |
| Kaempferol triglucoside | Flavonoids | Flavonols | 773.21 | 449.11 | 772.21 | [M+H]+ |
| Kaempferol-3,7-O-diglucoside | Flavonoids | Flavonols | 609.15 | 285.04 | 610.15 | [M-H]- |
| Kaempferol-3-O-(2''-O-acetyl)glucoside | Flavonoids | Flavonols | 491.12 | 287.05 | 490.11 | [M+H]+ |
| Kaempferol-3-O-(2''-O-xylosyl-6''-O-rhamnosyl)glucoside | Flavonoids | Flavonols | 727.21 | 287.05 | 726.20 | [M+H]+ |
| Kaempferol-3-O-(6'''-p-Coumaroyl)glucosyl-(1→2)-Glucoside-7-O-Rhamnoside | Flavonoids | Flavonols | 903.26 | 287.06 | 902.25 | [M+H]+ |
| Kaempferol-3-O-(6''-Acetyl)glucosyl-(1→3)-Galactoside | Flavonoids | Flavonols | 653.17 | 287.06 | 652.16 | [M+H]+ |
| Kaempferol-3-O-(6''-Rhamnosyl-2''-Glucosyl)Glucoside (Camelliaside A) | Flavonoids | Flavonols | 757.22 | 287.06 | 756.21 | [M+H]+ |
| Kaempferol-3-O-(6''-malonyl)galactoside | Flavonoids | Flavonols | 535.11 | 287.05 | 534.10 | [M+H]+ |
| Kaempferol-3-O-(6''-malonyl)glucoside | Flavonoids | Flavonols | 535.11 | 287.05 | 534.10 | [M+H]+ |
| Kaempferol-3-O-[2-O-(trans-p-coumaroyl)-3-O-ɑ-D-glucopyranosyl]-ɑ-D-glucopyranoside | Flavonoids | Flavonols | 755.18 | 285.04 | 756.19 | [M-H]- |
| Kaempferol-3-O-arabinoside | Flavonoids | Flavonols | 419.1 | 287.05 | 418.09 | [M+H]+ |
| Kaempferol-3-O-arabinoside (Juglanin) | Flavonoids | Flavonols | 419.1 | 287.06 | 418.09 | [M+H]+ |
| Kaempferol-3-O-arabinoside-7-O-rhamnoside | Flavonoids | Flavonols | 565.15 | 287.05 | 564.15 | [M+H]+ |
| Kaempferol-3-O-galactoside (Trifolin) | Flavonoids | Flavonols | 447.09 | 285.04 | 448.10 | [M-H]- |
| Kaempferol-3-O-galactoside-4'-O-glucoside | Flavonoids | Flavonols | 611.16 | 287.06 | 610.15 | [M+H]+ |
| Kaempferol-3-O-glucorhamnoside | Flavonoids | Flavonols | 595.17 | 287.06 | 594.16 | [M+H]+ |
| Kaempferol-3-O-glucoside (Astragalin) | Flavonoids | Flavonols | 449.11 | 287.06 | 448.10 | [M+H]+ |
| Kaempferol-3-O-glucoside-7-O-rhamnoside | Flavonoids | Flavonols | 595.17 | 287.06 | 594.16 | [M+H]+ |
| Kaempferol-3-O-mannoside（Amoenin） | Flavonoids | Flavonols | 449.11 | 287.06 | 448.10 | [M+H]+ |
| Kaempferol-3-O-neohesperidoside | Flavonoids | Flavonols | 595.16 | 287.06 | 594.16 | [M+H]+ |
| Kaempferol-3-O-rhamnosyl(1→2)glucoside | Flavonoids | Flavonols | 595.16 | 287.05 | 594.16 | [M+H]+ |
| Kaempferol-3-O-robinobioside(Biorobin) | Flavonoids | Flavonols | 593.15 | 285.04 | 594.16 | [M-H]- |
| Kaempferol-3-O-robinoside-7-O-rhamnoside (Robinin) | Flavonoids | Flavonols | 741.22 | 433.11 | 740.22 | [M+H]+ |
| Kaempferol-3-O-rutinoside(Nicotiflorin) | Flavonoids | Flavonols | 593.15 | 285.04 | 594.16 | [M-H]- |
| Kaempferol-3-O-rutinoside-7-O-glucoside | Flavonoids | Flavonols | 757.22 | 287.06 | 756.21 | [M+H]+ |
| Kaempferol-3-O-rutinoside-7-O-rhamnoside | Flavonoids | Flavonols | 741.22 | 287.06 | 740.22 | [M+H]+ |
| Kaempferol-3-O-sophoroside | Flavonoids | Flavonols | 609.15 | 285.04 | 610.15 | [M-H]- |
| Kaempferol-3-O-sophoroside-7-O-glucoside | Flavonoids | Flavonols | 773.21 | 287.06 | 772.21 | [M+H]+ |
| Kaempferol-3-O-sophorotrioside | Flavonoids | Flavonols | 773.21 | 449.11 | 772.21 | [M+H]+ |
| Kaempferol-3-O-sulfonate | Flavonoids | Flavonols | 365 | 285.04 | 366.00 | [M-H]- |
| Kaempferol-6,8-di-C-glucoside | Flavonoids | Flavonols | 611.16 | 491.12 | 610.15 | [M+H]+ |
| Kaempferol-6,8-di-C-glucoside-7-O-glucoside | Flavonoids | Flavonols | 773.21 | 287.06 | 772.21 | [M+H]+ |
| Kaempferol-7-O-glucoside | Flavonoids | Flavonols | 447.09 | 285.04 | 448.10 | [M-H]- |
| Kaempferol-7-O-rhamnoside | Flavonoids | Flavonols | 431.1 | 285.04 | 432.11 | [M-H]- |
| Kaempferol-xylosyl glucosyl glucoside | Flavonoids | Flavonols | 743.2 | 287.06 | 742.20 | [M+H]+ |
| Laricitrin-3-O-glucoside | Flavonoids | Flavonols | 495.11 | 333.07 | 494.11 | [M+H]+ |
| Morin | Flavonoids | Flavonols | 301.04 | 151 | 302.04 | [M-H]- |
| Morin-3-O-arabinoside | Flavonoids | Flavonols | 433.08 | 300.03 | 434.08 | [M-H]- |
| Morin-3-O-lyxoside | Flavonoids | Flavonols | 433.08 | 300.03 | 434.08 | [M-H]- |
| Morin-3-O-xyloside | Flavonoids | Flavonols | 435.09 | 303.05 | 434.08 | [M+H]+ |
| Myricetin | Flavonoids | Flavonols | 319.05 | 153.02 | 318.04 | [M+H]+ |
| Patuletin-3-O-glucoside | Flavonoids | Flavonols | 495.11 | 333.07 | 494.11 | [M+H]+ |
| Phellatin | Flavonoids | Flavonols | 533.17 | 371.11 | 534.17 | [M-H]- |
| Quercetin | Flavonoids | Flavonols | 303.05 | 229.05 | 302.04 | [M+H]+ |
| Quercetin 3-O-glucoside 7-O-xyloside | Flavonoids | Flavonols | 597.15 | 303.05 | 596.14 | [M+H]+ |
| Quercetin-3,4'-Dimethyl Ether | Flavonoids | Flavonols | 331.08 | 287.05 | 330.07 | [M+H]+ |
| Quercetin-3-O-(2''-O-arabinosyl)rutinoside | Flavonoids | Flavonols | 743.2 | 303.05 | 742.20 | [M+H]+ |
| Quercetin-3-O-(4''-O-glucosyl)rhamnoside | Flavonoids | Flavonols | 611.16 | 303.05 | 610.15 | [M+H]+ |
| Quercetin-3-O-(6''-O-p-Coumaroyl)glucoside | Flavonoids | Flavonols | 611.14 | 147.04 | 610.13 | [M+H]+ |
| Quercetin-3-O-alloside; Isohyperoside | Flavonoids | Flavonols | 465.1 | 303.06 | 464.10 | [M+H]+ |
| Quercetin-3-O-arabinoside | Flavonoids | Flavonols | 433.08 | 300.03 | 434.08 | [M-H]- |
| Quercetin-3-O-galactoside (Hyperin) | Flavonoids | Flavonols | 465.1 | 303.06 | 464.10 | [M+H]+ |
| Quercetin-3-O-glucoside (Isoquercitrin) | Flavonoids | Flavonols | 465.1 | 303.05 | 464.10 | [M+H]+ |
| Quercetin-3-O-rhamnoside(Quercitrin) | Flavonoids | Flavonols | 449.11 | 303.05 | 448.10 | [M+H]+ |
| Quercetin-3-O-robinobioside | Flavonoids | Flavonols | 609.15 | 300.03 | 610.15 | [M-H]- |
| Quercetin-3-O-rutinoside (Rutin) | Flavonoids | Flavonols | 611.16 | 303.05 | 610.15 | [M+H]+ |
| Quercetin-3-O-rutinoside-7-O-rhamnoside | Flavonoids | Flavonols | 757.21 | 303.05 | 756.21 | [M+H]+ |
| Quercetin-3-O-sophoroside-7-O-rhamnoside | Flavonoids | Flavonols | 773.21 | 303.05 | 772.21 | [M+H]+ |
| Quercetin-3-O-xyloside (Reynoutrin) | Flavonoids | Flavonols | 435.09 | 303.06 | 434.08 | [M+H]+ |
| Quercetin-3-O-α-rhamnosyl (1→2)-[α-rhamnosyl (1→6)]-β-glucoside | Flavonoids | Flavonols | 757.22 | 303.05 | 756.21 | [M+H]+ |
| Quercetin-4'-O-glucoside (Spiraeoside) | Flavonoids | Flavonols | 463.09 | 301.04 | 464.10 | [M-H]- |
| Quercetin-5-O-β-D-glucoside | Flavonoids | Flavonols | 465.1 | 303.05 | 464.10 | [M+H]+ |
| Quercetin-7-O-glucoside | Flavonoids | Flavonols | 463.09 | 301.04 | 464.10 | [M-H]- |
| Quercetin-7-O-rutinoside | Flavonoids | Flavonols | 611.16 | 303.05 | 610.15 | [M+H]+ |
| Quercetin-7-O-rutinoside-4'-O-glucoside | Flavonoids | Flavonols | 773.21 | 303.05 | 772.21 | [M+H]+ |
| Rhamnazin (3,4',5-Trihydroxy-3',7-dimethoxyflavone) | Flavonoids | Flavonols | 331.08 | 167.03 | 330.07 | [M+H]+ |
| Rhamnetin-3-O-Glucoside | Flavonoids | Flavonols | 479.12 | 317.07 | 478.11 | [M+H]+ |
| Rhamnetin-3-O-Rutinoside | Flavonoids | Flavonols | 625.1661 | 317.066 | 624.17 | [M+H]+ |
| Rhamnetin-3-O-Rutinoside-5-O-rhamnoside | Flavonoids | Flavonols | 771.22 | 317.06 | 770.23 | [M+H]+ |
| Rutin Trihydrate | Flavonoids | Flavonols | 611.16 | 303.06 | 664.19 | [M-3H2O+H]+ |
| Sexangularetin-3-O-glucoside-7-O-rhamnoside | Flavonoids | Flavonols | 625.18 | 317.07 | 624.17 | [M+H]+ |
| Tamarixetin (3,3',5,7-Tetrahydroxy-4'-Methoxyflavone) | Flavonoids | Flavonols | 317.07 | 285.04 | 316.06 | [M+H]+ |
| Tamarixetin-3-O-rutinoside | Flavonoids | Flavonols | 623.16 | 315.05 | 624.17 | [M-H]- |
| kaempferol-3-O-(2-glucosylrutinoside)-7-O-glucoside | Flavonoids | Flavonols | 919.27 | 287.06 | 918.26 | [M+H]+ |
| kaempferol-3-O-(2-xylosyl rutinoside)-7-O-glucoside | Flavonoids | Flavonols | 889.26 | 287.06 | 888.25 | [M+H]+ |
| kaempferol-3-O-(acetyl rutinoside)-7-O-glucoside | Flavonoids | Flavonols | 799.23 | 287.06 | 798.22 | [M+H]+ |
| kaempferol-3-O-β-glucopyranosyl-(1→2)-[α-rhamnopyranosyl (1→6)]-β-galactopyranoside-7-O-α-rhamnopyranoside | Flavonoids | Flavonols | 903.27 | 433.11 | 902.27 | [M+H]+ |
| kaempferol-3-p-coumaroyldiglucoside | Flavonoids | Flavonols | 755.19 | 285.04 | 756.19 | [M-H]- |
| kaempferol-3-p-coumaroyldiglucoside-7-glucoside | Flavonoids | Flavonols | 917.24 | 755.19 | 918.24 | [M-H]- |
| 2-Hydroxy-2,3-dihydrogenistein | Flavonoids | Isoflavones | 287.06 | 125.02 | 288.06 | [M-H]- |
| 3'-Methoxydaidzin | Flavonoids | Isoflavones | 447.13 | 285.08 | 446.12 | [M+H]+ |
| 3'-hydroxyPuerarin | Flavonoids | Isoflavones | 433.11 | 313.07 | 432.11 | [M+H]+ |
| 3,9-Dihydroxypterocarpan | Flavonoids | Isoflavones | 255.07 | 119.05 | 256.07 | [M-H]- |
| 4',6,7-Trihydroxyisoflavone | Flavonoids | Isoflavones | 271.06 | 215.07 | 270.05 | [M+H]+ |
| 7-Hydroxy-3'-methoxy-isoflavone-7-primeveroside | Flavonoids | Isoflavones | 561.16 | 267.07 | 562.17 | [M-H]- |
| Aracarpene 1 | Flavonoids | Isoflavones | 299.06 | 284.02 | 300.06 | [M-H]- |
| Aracarpene 2 | Flavonoids | Isoflavones | 299.06 | 284.02 | 300.06 | [M-H]- |
| Biochanin A-7-O-glucoside (Sissotrin) | Flavonoids | Isoflavones | 447.13 | 285.07 | 446.12 | [M+H]+ |
| Cajanin | Flavonoids | Isoflavones | 301.07 | 258.05 | 300.06 | [M+H]+ |
| Cernuoside | Flavonoids | Isoflavones | 449.11 | 317.07 | 448.10 | [M+H]+ |
| Derriscandenoside A | Flavonoids | Isoflavones | 447.13 | 285.08 | 446.12 | [M+H]+ |
| Ferreirin | Flavonoids | Isoflavones | 301.07 | 135.04 | 302.08 | [M-H]- |
| Genistein | Flavonoids | Isoflavones | 271.06 | 153.02 | 270.05 | [M+H]+ |
| Genistein-7-O-galactoside | Flavonoids | Isoflavones | 433.11 | 271.06 | 432.11 | [M+H]+ |
| Genistein-8-C-glucoside-O-apiosyl | Flavonoids | Isoflavones | 565.16 | 433.11 | 564.15 | [M+H]+ |
| Iristectorigenin A | Flavonoids | Isoflavones | 331.0835 | 316.05 | 330.07 | [M+H]+ |
| Iristectorin A | Flavonoids | Isoflavones | 493.13 | 331.08 | 492.13 | [M+H]+ |
| Isoluteolin (Orobol)(5,7,3',4'-tetrahydroxyisoflavone) | Flavonoids | Isoflavones | 287.06 | 241.05 | 286.05 | [M+H]+ |
| Isoluteolin-6,8-di-C-glucoside | Flavonoids | Isoflavones | 611.16 | 491.12 | 610.15 | [M+H]+ |
| Maackiain-3-O-glucosyl-6''-O-malonate | Flavonoids | Isoflavones | 533.13 | 285.08 | 532.12 | [M+H]+ |
| Medicarpin-3-O-Glucoside; Medicocarpin | Flavonoids | Isoflavones | 433.15 | 271.09 | 432.14 | [M+H]+ |
| Medicarpinglucoside | Flavonoids | Isoflavones | 433.15 | 271.1 | 432.14 | [M+H]+ |
| Prunetin (5,4'-Dihydroxy-7-methoxyisoflavone) | Flavonoids | Isoflavones | 283.06 | 268.04 | 284.07 | [M-H]- |
| Prunetin-4'-O-glucoside(Prunitrin) | Flavonoids | Isoflavones | 447.13 | 285.08 | 446.12 | [M+H]+ |
| Prunetin-5-O-glucoside | Flavonoids | Isoflavones | 447.13 | 285.07 | 446.12 | [M+H]+ |
| Sophoricoside | Flavonoids | Isoflavones | 433.11 | 271.06 | 432.11 | [M+H]+ |
| Tectorigenin | Flavonoids | Isoflavones | 299.06 | 284.03 | 300.06 | [M-H]- |
| paratensein-7-O-glucoside | Flavonoids | Isoflavones | 463.12 | 301.07 | 462.12 | [M+H]+ |
| sophorabioside | Flavonoids | Isoflavones | 579.17 | 271.06 | 578.16 | [M+H]+ |
| (+)-epipinoresinol | Lignans and Coumarins | Lignans | 357.13 | 151.04 | 358.14 | [M-H]- |
| (1R,2S)-2-[4-[(3R,3aR,6R,6aS)-3-(4-Hydroxy-3,5-dimethoxyphenyl)-1,3,3a,4,6,6a-hexahydrofuro[3,4-c]furan-6-yl]-2,6-dimethoxyphenoxy]-1-(4-hydroxy-3,5-dimethoxyphenyl)propane-1,3-diol | Lignans and Coumarins | Lignans | 643.24 | 595.22 | 644.25 | [M-H]- |
| (2S,3R,4S,5S,6R)-2-[4-[(1R,2R)-2-[4-[(3R,3aS,6R,6aS)-3-(4-hydroxy-3,5-dimethoxyphenyl)-1,3,3a,4,6,6a-hexahydrofuro[3,4-c]furan-6-yl]-2,6-dimethoxyphenoxy]-1,3-dihydroxypropyl]-2,6-dimethoxyphenoxy]-6-(hydroxymethyl)oxane-3,4,5-triol | Lignans and Coumarins | Lignans | 805.29 | 583.22 | 806.30 | [M-H]- |
| (2S,3R,4S,5S,6R)-2-[4-[(3R,3aS,6S,6aS)-6-[4-[(1R,2S)-1-[3,5-Dimethoxy-4-[(2S,3R,4S,5S,6R)-3,4,5-trihydroxy-6-(hydroxymethyl)oxan-2-yl]oxyphenyl]-1,3-dihydroxypropan-2-yl]oxy-3,5-dimethoxyphenyl]-1,3,3a,4,6,6a-hexahydrofuro[3,4-c]furan-3-yl]-2,6-dimethoxyphenoxy]-6-(hydroxymethyl)oxane-3,4,5-triol | Lignans and Coumarins | Lignans | 967.34 | 745.27 | 968.35 | [M-H]- |
| (2S,3R,4S,6S)-2-[4-[(1R,2R)-1,3-Dihydroxy-2-[4-[(E)-3-hydroxyprop-1-enyl]-2,6-dimethoxyphenoxy]propyl]-2,6-dimethoxyphenoxy]-6-(hydroxymethyl)oxane-3,4-diol | Lignans and Coumarins | Lignans | 581.22 | 329.14 | 582.23 | [M-H]- |
| (2r,3r,4s)-6-hydroxy-4-(4-hydroxy-3-methoxyphenyl)-7-methoxy-2,3-dimethyl-3,4-dihydro-2h-naphthalen-1-one | Lignans and Coumarins | Lignans | 341.14 | 311.09 | 342.15 | [M-H]- |
| (3r,4r)-3-(2h-1,3-benzodioxol-5-ylmethyl)-4-[(4-hydroxy-3-methoxyphenyl)methyl]oxolan-2-one | Lignans and Coumarins | Lignans | 357.13 | 307.1 | 356.13 | [M+H]+ |
| (7'R,8'R)-7'8'-dihydro-7'-(5'-hydroxy-3'-methoxphenyl)-3-methoxy-8'-methyl-1-(E)-propenylbenzof-uran | Lignans and Coumarins | Lignans | 327.16 | 137.06 | 326.15 | [M+H]+ |
| (7'S,8R,8'R)-3,5'-dimethoxy-3',4,9'-trihydroxy-7',9-epoxy-8,8'-lignan | Lignans and Coumarins | Lignans | 359.15 | 329.14 | 360.16 | [M-H]- |
| (7R,8S)-9-acetyl-dehydrodiconiferyl alcohol | Lignans and Coumarins | Lignans | 401.16 | 217.09 | 400.15 | [M+H]+ |
| 1,4-Benzodioxin-6-propanol | Lignans and Coumarins | Lignans | 193.09 | 105.07 | 192.08 | [M+H]+ |
| 1-(4-Hydroxy-3-Methoxyphenyl)-2-(4-(3-Hydroxypropyl)-2,6-Dimethoxyphenoxy)Propane-1,3-Diol | Lignans and Coumarins | Lignans | 407.17 | 359.15 | 408.18 | [M-H]- |
| 1-(4-Hydroxy-3-methoxyphenyl)-2-[4-(1,2,3-trihydroxypropyl)-2-methoxyphenoxy]-1,3-propanediol* | Lignans and Coumarins | Lignans | 409.15 | 165.06 | 410.16 | [M-H]- |
| 1-Hydroxypinoresinol-1-O-Glucoside | Lignans and Coumarins | Lignans | 535.18 | 373.13 | 536.19 | [M-H]- |
| 3,3'-Bis(3,4-dihydro-4-hydroxy-6,8-dimethoxy-2H-1-benzopyran) | Lignans and Coumarins | Lignans | 417.16 | 181.05 | 418.16 | [M+H]+ |
| 3,4,5,19-tetramethoxy-9,10-dimethyl-15,17-dioxatetracyclo[10.7.0.02,7.014,18]nonadeca-1(19),2,4,6,12,14(18)-hexaen-11-ol | Lignans and Coumarins | Lignans | 417.15 | 167.07 | 416.15 | [M+H]+ |
| 3,4-Dihydro-4-(4-hydroxy-3-methoxyphenyl)-3-(hydroxymethyl)-6,7-dimethoxy-(3R,4S)-2-naphthalenecarboxaldehyde | Lignans and Coumarins | Lignans | 369.13 | 339.09 | 370.14 | [M-H]- |
| 3,4-Divanillyltetrahydrofuran | Lignans and Coumarins | Lignans | 345.17 | 137.06 | 344.16 | [M+H]+ |
| 4',8''-Bisguaiacylglycerol | Lignans and Coumarins | Lignans | 409.15 | 165.06 | 410.16 | [M-H]- |
| 5'-Demethylaquillochin | Lignans and Coumarins | Lignans | 387.11 | 191.07 | 386.10 | [M+H]+ |
| 5'-Methoxyisolariciresinol-9'-O-glucoside | Lignans and Coumarins | Lignans | 551.21 | 389.17 | 552.22 | [M-H]- |
| 5'-Methoxymatairesinoside | Lignans and Coumarins | Lignans | 549.2 | 387.15 | 550.21 | [M-H]- |
| 5-[4-(3,4-dimethoxyphenyl)-hexahydrofuro[3,4-c]furan-1-yl]-4-methyl-2H-1,3-benzodioxole | Lignans and Coumarins | Lignans | 385.16 | 206.08 | 384.16 | [M+H]+ |
| 7,8-Dihydro-Buddlenol B(threo) | Lignans and Coumarins | Lignans | 585.23 | 537.21 | 586.24 | [M-H]- |
| 8,8''-Bisguaiacylglycerol | Lignans and Coumarins | Lignans | 409.15 | 165.06 | 410.16 | [M-H]- |
| 8-Hydroxy-α-conidendrin | Lignans and Coumarins | Lignans | 373.12 | 105.03 | 372.12 | [M+H]+ |
| 8R-Dihydrodehydrodiconferyl alcohol 4-O-β-D-glucopyranoside | Lignans and Coumarins | Lignans | 507.18 | 161.02 | 508.19 | [M-H]- |
| Acanthoside D | Lignans and Coumarins | Lignans | 741.26 | 417.15 | 742.27 | [M-H]- |
| Aglacin F | Lignans and Coumarins | Lignans | 447.2 | 219.1 | 446.19 | [M+H]+ |
| Anhydrosecoisolariciresinol (AHS) | Lignans and Coumarins | Lignans | 345.17 | 137.06 | 344.16 | [M+H]+ |
| Arctigenin | Lignans and Coumarins | Lignans | 373.17 | 137.06 | 372.16 | [M+H]+ |
| Balanophonin E glucoside | Lignans and Coumarins | Lignans | 551.21 | 193.09 | 550.21 | [M+H]+ |
| Buddlenol F glucoside | Lignans and Coumarins | Lignans | 775.28 | 613.23 | 776.29 | [M-H]- |
| Cagayanone A | Lignans and Coumarins | Lignans | 337.11 | 307.06 | 338.12 | [M-H]- |
| Ciwujiatone | Lignans and Coumarins | Lignans | 435.16 | 181.05 | 434.16 | [M+H]+ |
| Cycloolivil-6-O-glucoside | Lignans and Coumarins | Lignans | 537.2 | 375.15 | 538.21 | [M-H]- |
| Dehydrodiconiferyl alcohol-4-O-glucoside | Lignans and Coumarins | Lignans | 521.2 | 131.05 | 520.19 | [M+H]+ |
| Dehydrodiconiferyl alcohol-gamma'-O-glucoside | Lignans and Coumarins | Lignans | 521.2 | 131.05 | 520.19 | [M+H]+ |
| Dehydrodiconiferylalcohol-9'-O-glucoside | Lignans and Coumarins | Lignans | 519.19 | 357.13 | 520.19 | [M-H]- |
| Demethyl-Secoisolariciresinol | Lignans and Coumarins | Lignans | 347.15 | 165.05 | 348.16 | [M-H]- |
| Denudatone | Lignans and Coumarins | Lignans | 387.18 | 299.12 | 386.17 | [M+H]+ |
| Dichotomoside A | Lignans and Coumarins | Lignans | 551.18 | 193.05 | 552.18 | [M-H]- |
| Dihydrodehydrodiconiferyl alcohol-4-O-glucoside | Lignans and Coumarins | Lignans | 521.2 | 359.15 | 522.21 | [M-H]- |
| Dimethylmatairesinol | Lignans and Coumarins | Lignans | 387.18 | 151.08 | 386.17 | [M+H]+ |
| Divanillyltetrahydrofuran | Lignans and Coumarins | Lignans | 345.17 | 137.06 | 344.16 | [M+H]+ |
| Eleutheroside E | Lignans and Coumarins | Lignans | 741.26 | 579.21 | 742.27 | [M-H]- |
| Enterodiol | Lignans and Coumarins | Lignans | 301.14 | 253.12 | 302.15 | [M-H]- |
| Epipinoresinol | Lignans and Coumarins | Lignans | 357.13 | 151.04 | 358.14 | [M-H]- |
| Erythro-Guaiacylglycerol-β-Coniferyl Ether | Lignans and Coumarins | Lignans | 375.15 | 327.12 | 376.15 | [M-H]- |
| Erythro-Guaiacylglycerol-β-O-4'-dehydrodisinapyl Ether | Lignans and Coumarins | Lignans | 583.22 | 535.2 | 584.23 | [M-H]- |
| Erythro-Guaiacylglycerol-β-Sinapyl Ether | Lignans and Coumarins | Lignans | 405.15 | 165.06 | 406.16 | [M-H]- |
| Erythro-Guaiacylglycerol-β-dihydroconiferyl Ether | Lignans and Coumarins | Lignans | 377.16 | 329.14 | 378.17 | [M-H]- |
| Erythro-Guaiacylglycerol-β-dihydroconiferyl Ether glucoside | Lignans and Coumarins | Lignans | 539.21 | 491.19 | 540.22 | [M-H]- |
| Erythro-Guaiacylglycerol-β-threo-syringylglycerol Ether | Lignans and Coumarins | Lignans | 439.16 | 195.07 | 440.17 | [M-H]- |
| Erythro-Guaiacylglycerol-β-threo-syringylglycerol Ether glucoside | Lignans and Coumarins | Lignans | 601.21 | 439.16 | 602.22 | [M-H]- |
| Fargesin | Lignans and Coumarins | Lignans | 371.15 | 167.07 | 370.14 | [M+H]+ |
| Ficusesquilignan B | Lignans and Coumarins | Lignans | 583.22 | 195.07 | 584.23 | [M-H]- |
| Forsythialan B | Lignans and Coumarins | Lignans | 389.16 | 165.05 | 388.15 | [M+H]+ |
| Fraxiresinol | Lignans and Coumarins | Lignans | 405.15 | 167.07 | 404.15 | [M+H]+ |
| Guaiacylglycerol-β-Guaiacyl Ether | Lignans and Coumarins | Lignans | 319.12 | 241.05 | 320.13 | [M-H]- |
| Icariside E5 | Lignans and Coumarins | Lignans | 521.2 | 359.15 | 522.21 | [M-H]- |
| Isolariciresinol | Lignans and Coumarins | Lignans | 359.15 | 180.08 | 360.16 | [M-H]- |
| Isolariciresinol-9'-O-glucoside | Lignans and Coumarins | Lignans | 521.2 | 359.15 | 522.21 | [M-H]- |
| Justicidin A | Lignans and Coumarins | Lignans | 395.11 | 365.06 | 394.11 | [M+H]+ |
| Justicidin C | Lignans and Coumarins | Lignans | 395.11 | 365.07 | 394.11 | [M+H]+ |
| Lariciresinol | Lignans and Coumarins | Lignans | 361.16 | 189.09 | 360.16 | [M+H]+ |
| Lariciresinol-4'-O-glucoside | Lignans and Coumarins | Lignans | 521.2 | 359.15 | 522.21 | [M-H]- |
| Ligraminol E | Lignans and Coumarins | Lignans | 361.16 | 181.09 | 362.17 | [M-H]- |
| Lirioresinol A | Lignans and Coumarins | Lignans | 417.15 | 181.04 | 418.16 | [M-H]- |
| Lyoniresinol-3α-O-glucoside | Lignans and Coumarins | Lignans | 581.22 | 401.16 | 582.23 | [M-H]- |
| Machilin H | Lignans and Coumarins | Lignans | 375.18 | 177.09 | 374.17 | [M+H]+ |
| Magnolin | Lignans and Coumarins | Lignans | 417.19 | 151.07 | 416.18 | [M+H]+ |
| Manglieside D | Lignans and Coumarins | Lignans | 521.2 | 329.14 | 522.21 | [M-H]- |
| Massoniresinol; Vladinol A | Lignans and Coumarins | Lignans | 393.15 | 151.04 | 392.15 | [M+H]+ |
| Matairesinol | Lignans and Coumarins | Lignans | 359.15 | 137.06 | 358.14 | [M+H]+ |
| Matairesinol-4'-O-glucoside (Matairesinoside) | Lignans and Coumarins | Lignans | 521.2 | 131.05 | 520.19 | [M+H]+ |
| Matairesinol-4,4'-O-diglucoside | Lignans and Coumarins | Lignans | 681.24 | 357.13 | 682.25 | [M-H]- |
| Medioresinol-4,4'-di-O-glucoside | Lignans and Coumarins | Lignans | 711.25 | 549.2 | 712.26 | [M-H]- |
| Nortrachelogenin-4-O-glucoside | Lignans and Coumarins | Lignans | 535.18 | 373.13 | 536.19 | [M-H]- |
| Olivil Monoacetate | Lignans and Coumarins | Lignans | 419.17 | 205.09 | 418.16 | [M+H]+ |
| Olivil-4'-O-glucoside | Lignans and Coumarins | Lignans | 537.2 | 375.15 | 538.21 | [M-H]- |
| Phillygenin | Lignans and Coumarins | Lignans | 373.16 | 337.14 | 372.16 | [M+H]+ |
| Picraquassioside C | Lignans and Coumarins | Lignans | 597.22 | 375.14 | 598.23 | [M-H]- |
| Pinoresinol dimethyl ether | Lignans and Coumarins | Lignans | 387.18 | 151.08 | 386.17 | [M+H]+ |
| Pinoresinol | Lignans and Coumarins | Lignans | 357.13 | 151.04 | 358.14 | [M-H]- |
| Pinoresinol-4-O-glucoside | Lignans and Coumarins | Lignans | 519.19 | 357.14 | 520.19 | [M-H]- |
| Saikolignanoside A | Lignans and Coumarins | Lignans | 521.2 | 329.14 | 522.21 | [M-H]- |
| Secoisolariciresinol | Lignans and Coumarins | Lignans | 361.17 | 180.08 | 362.17 | [M-H]- |
| Secoisolariciresinol 4-O-glucoside | Lignans and Coumarins | Lignans | 523.22 | 361.14 | 524.23 | [M-H]- |
| Sesamolinol-glucoside | Lignans and Coumarins | Lignans | 533.17 | 371.11 | 534.17 | [M-H]- |
| Symplocosin | Lignans and Coumarins | Lignans | 521.2 | 131.05 | 520.19 | [M+H]+ |
| Syringaresinol | Lignans and Coumarins | Lignans | 417.16 | 402.1 | 418.16 | [M-H]- |
| Syringaresinol-4'-O-(6''-acetyl)glucoside | Lignans and Coumarins | Lignans | 621.22 | 417.16 | 622.23 | [M-H]- |
| Syringaresinol-4'-O-glucoside | Lignans and Coumarins | Lignans | 579.2 | 339.12 | 580.22 | [M-H]- |
| Syringaresinol-4'-O-glucoside; Acanthoside B | Lignans and Coumarins | Lignans | 579.21 | 417.16 | 580.22 | [M-H]- |
| Tortoside B | Lignans and Coumarins | Lignans | 581.22 | 329.14 | 582.23 | [M-H]- |
| Tracheloside | Lignans and Coumarins | Lignans | 549.2 | 387.14 | 550.21 | [M-H]- |
| Verrucosin | Lignans and Coumarins | Lignans | 345.17 | 137.06 | 344.16 | [M+H]+ |
| Vladinol D | Lignans and Coumarins | Lignans | 375.14 | 151.04 | 374.14 | [M+H]+ |
| [(1R,2S)-1-(1,3-benzodioxol-5-yl)-2-methyl-3-oxobutyl]4-hydroxy-3-methoxybenzoate | Lignans and Coumarins | Lignans | 373.13 | 343.08 | 372.12 | [M+H]+ |
| [(2S)-2α-[3,5-Dimethoxy-4-[[(αS,βR)-α-(Hydroxymethyl)-β,4-Dihydroxy-3-Methoxyphenethyl]Oxy]Phenyl]-5-(3-Hydroxy-1-Propenyl)-7-Methoxy-2,3-Dihydrobenzofuran]-3β-Ylmethylβ-D-Glucopyranoside | Lignans and Coumarins | Lignans | 745.27 | 583.22 | 746.28 | [M-H]- |
| [(2S,3S)-2-(3,4-dimethoxyphenyl)-7-methoxy-5-prop-2-enyl-2,3-dihydro-1-benzofuran-3-yl]methanol | Lignans and Coumarins | Lignans | 357.17 | 151.07 | 356.16 | [M+H]+ |
| alaschanioside C | Lignans and Coumarins | Lignans | 519.19 | 339.12 | 520.19 | [M-H]- |
| demethoxypinoresinol | Lignans and Coumarins | Lignans | 327.12 | 121.03 | 328.13 | [M-H]- |
| dihydrodehydrodiconiferyl alcohol-9-O-β-D-glucopyranoside | Lignans and Coumarins | Lignans | 521.2 | 491.19 | 522.21 | [M-H]- |
| dihydrosesamin | Lignans and Coumarins | Lignans | 357.13 | 307.09 | 356.13 | [M+H]+ |
| epieudesmin | Lignans and Coumarins | Lignans | 387.18 | 137.06 | 386.17 | [M+H]+ |
| guaiacylglycerol-β-coniferyl ether | Lignans and Coumarins | Lignans | 377.16 | 163.08 | 376.15 | [M+H]+ |
| isatioxyneolignoside E | Lignans and Coumarins | Lignans | 597.22 | 327.12 | 598.23 | [M-H]- |
| ligballinol | Lignans and Coumarins | Lignans | 297.11 | 121.03 | 298.12 | [M-H]- |
| saikolignanoside D | Lignans and Coumarins | Lignans | 521.2 | 131.05 | 520.19 | [M+H]+ |
| syringaresinol-4-O-β-D-glucopyranoside | Lignans and Coumarins | Lignans | 579.21 | 417.16 | 580.22 | [M-H]- |
| tetrahydrofuroguaiacin B | Lignans and Coumarins | Lignans | 345.17 | 137.06 | 344.16 | [M+H]+ |
| tortoside f | Lignans and Coumarins | Lignans | 519.18 | 339.12 | 518.18 | [M+H]+ |
| trans-1,2-dihydrodehydroguaiaretic acid | Lignans and Coumarins | Lignans | 327.16 | 151.08 | 326.15 | [M+H]+ |
| 1,2,4,5,8-pentahydroxy-6-methylanthracene-9,10-dione | Flavonoids | Other Flavonoids | 301.04 | 151 | 302.04 | [M-H]- |
| 1,2,5,7,8-pentahydroxy-3-methylanthracene-9,10-dione | Flavonoids | Other Flavonoids | 303.05 | 303.05 | 302.04 | [M+H]+ |
| 1,3,6,7-tetrahydroxy-2-(3,4,5-trihydroxyoxan-2-yl)xanthen-9-one | Flavonoids | Other Flavonoids | 391.07 | 229.05 | 392.07 | [M-H]- |
| 1,3,7-trihydroxy-2-[3,4,5-trihydroxy-6-(hydroxymethyl)oxan-2-yl]xanthen-9-one | Flavonoids | Other Flavonoids | 405.08 | 243.03 | 406.09 | [M-H]- |
| 1,6-dihydroxy-3,7-dimethoxy-8-(3-methoxy-3-methylbutyl)-2-(3-methylbut-2-en-1-yl)xanthen-9-one | Flavonoids | Other Flavonoids | 455.21 | 395.19 | 456.21 | [M-H]- |
| 1,8-dihydroxy-2,6-dimethylxanthen-9-one | Flavonoids | Other Flavonoids | 255.07 | 255.06 | 256.07 | [M-H]- |
| 1,8-dihydroxy-4,5-dimethoxy-3-{[(2s,3r,4s,5s,6r)-3,4,5-trihydroxy-6-(hydroxymethyl)oxan-2-yl]oxy}xanthen-9-one | Flavonoids | Other Flavonoids | 465.1 | 303.05 | 466.11 | [M-H]- |
| 2',7-Dihydroxy-3',4'-dimethoxyisoflavan | Flavonoids | Other Flavonoids | 303.12 | 195.09 | 302.12 | [M+H]+ |
| 2-hydroxy-8-methoxy-6-methyl-9-oxoxanthene-1-carboxylic acid | Flavonoids | Other Flavonoids | 299.05 | 284.03 | 300.06 | [M-H]- |
| 3'-Hydroxy-4'-O-methylglabridin | Flavonoids | Other Flavonoids | 353.14 | 295.06 | 354.15 | [M-H]- |
| 3,4,5,3'-tetrahydroxybenzophenone | Flavonoids | Other Flavonoids | 247.06 | 153.02 | 246.05 | [M+H]+ |
| 4'-Demethyleucomin glucoside | Flavonoids | Other Flavonoids | 447.13 | 285.07 | 446.12 | [M+H]+ |
| 4'-Demethyleucomin glucosyl rhamnoside | Flavonoids | Other Flavonoids | 593.18 | 285.07 | 592.18 | [M+H]+ |
| 4-C-Glucose-1,3,6-trihydroxy-7-methoxyxanthone | Flavonoids | Other Flavonoids | 435.09 | 345 | 436.10 | [M-H]- |
| 5,7-Dihydroxy-8-methyl-3-(2',4'-dihydroxybenzyl)-chroman-4-one | Flavonoids | Other Flavonoids | 315.09 | 125.02 | 316.09 | [M-H]- |
| 5-hydroxy-7-(1',2'-dihydroxypropyl)-2-methyl-chromone | Flavonoids | Other Flavonoids | 251.09 | 191.03 | 250.08 | [M+H]+ |
| 6,7-dihydroxy-1,3-dimethoxyxanthen-9-one | Flavonoids | Other Flavonoids | 287.05 | 135.04 | 288.06 | [M-H]- |
| 7,8-Dihydroxy-4'-methoxyisoflavone | Flavonoids | Other Flavonoids | 285.08 | 253.05 | 284.07 | [M+H]+ |
| 7-O-β-D-glucopyranoside-5-hydroxy-3-(4'-hydroxybenzylidene)-chroman-4-one | Flavonoids | Other Flavonoids | 447.13 | 285.08 | 446.12 | [M+H]+ |
| 8,11-dimethoxy-2h-[1,3]dioxolo[4,5-b]xanthen-10-one | Flavonoids | Other Flavonoids | 301.07 | 286.05 | 300.06 | [M+H]+ |
| 9,11-dimethoxy-2h-[1,3]dioxolo[4,5-b]xanthen-10-one | Flavonoids | Other Flavonoids | 299.06 | 284.03 | 300.06 | [M-H]- |
| Calyxanthone | Flavonoids | Other Flavonoids | 285.04 | 285.04 | 286.05 | [M-H]- |
| Drimiopsin C | Flavonoids | Other Flavonoids | 287.05 | 125.02 | 288.06 | [M-H]- |
| Lancerin; 4-C-Glucosyl-1,3,7-Trihydroxyxanthone | Flavonoids | Other Flavonoids | 405.08 | 243.03 | 406.09 | [M-H]- |
| Maesopsin | Flavonoids | Other Flavonoids | 287.05 | 125.03 | 288.06 | [M-H]- |
| Mangiferin | Flavonoids | Other Flavonoids | 421.08 | 301.05 | 422.08 | [M-H]- |
| sappanol | Flavonoids | Other Flavonoids | 305.1 | 139.04 | 304.09 | [M+H]+ |
| 1,3-O-Dicaffeoylglycerol | Phenolic acids | Phenolic acids | 417.12 | 163.04 | 416.11 | [M+H]+ |
| 1,6-Di-O-galloyl-β-D-glucose | Phenolic acids | Phenolic acids | 483.078 | 169.0143 | 484.09 | [M-H]- |
| 1,7-bis(4-hydroxy-3-methoxyphenyl)hept-1-ene-3-ol | Phenolic acids | Phenolic acids | 359.18 | 137.06 | 358.18 | [M+H]+ |
| 1,7-bis(4-hydroxy-3-methoxyphenyl)hept-6-ene-3-one | Phenolic acids | Phenolic acids | 357.17 | 137.06 | 356.16 | [M+H]+ |
| 1-(4-Hydroxybenzoyl)Glucose; 25545-07-7 | Phenolic acids | Phenolic acids | 299.08 | 93.03 | 300.08 | [M-H]- |
| 1-Feruloyl-sn-glycerol | Phenolic acids | Phenolic acids | 269.1 | 177.05 | 268.09 | [M+H]+ |
| 1-O-(3,4,5-Trimethoxybenzoyl)-B-D-Glucopyranoside | Phenolic acids | Phenolic acids | 373.11 | 211.06 | 374.12 | [M-H]- |
| 1-O-(3,4-Dihydroxy-5-methoxy-benzoyl)-glucoside | Phenolic acids | Phenolic acids | 345.08 | 139.04 | 346.09 | [M-H]- |
| 1-O-(p-coumaroyl) 3-Hydroxy-3-methylglutaric acid | Phenolic acids | Phenolic acids | 309.1 | 147.04 | 308.09 | [M+H]+ |
| 1-O-Caffeoyl-(6-O-glucosyl)-β-D-glucose | Phenolic acids | Phenolic acids | 503.14 | 341.09 | 504.15 | [M-H]- |
| 1-O-Caffeoyl-β-D-glucose | Phenolic acids | Phenolic acids | 341.09 | 179.04 | 342.10 | [M-H]- |
| 1-O-Caffeoyl-β-D-xylose | Phenolic acids | Phenolic acids | 311.08 | 163.04 | 312.08 | [M-H]- |
| 1-O-Galloyl-β-D-glucose | Phenolic acids | Phenolic acids | 333.08 | 171.03 | 332.07 | [M+H]+ |
| 1-O-Gentisoyl-β-D-glucoside | Phenolic acids | Phenolic acids | 315.07 | 153.02 | 316.08 | [M-H]- |
| 1-O-Salicyloyl-β-D-glucose | Phenolic acids | Phenolic acids | 299.08 | 137.02 | 300.08 | [M-H]- |
| 1-O-Sinapoyl-β-D-glucose | Phenolic acids | Phenolic acids | 385.11 | 223.06 | 386.12 | [M-H]- |
| 1-O-p-Coumaroyl Galactonic Acid | Phenolic acids | Phenolic acids | 341.09 | 195.05 | 342.10 | [M-H]- |
| 1-O-p-Coumaroyl-β-D-glucose | Phenolic acids | Phenolic acids | 325.09 | 163.04 | 326.10 | [M-H]- |
| 1-O-p-Coumaroylquinic acid | Phenolic acids | Phenolic acids | 337.09 | 163.04 | 338.10 | [M-H]- |
| 10-acetylmajoroside | Phenolic acids | Phenolic acids | 445.13 | 241.07 | 446.14 | [M-H]- |
| 2',4'-Dimethoxyacetophenone | Phenolic acids | Phenolic acids | 181.08 | 139.04 | 180.08 | [M+H]+ |
| 2'-Hydroxy-4'-Methylacetophenone | Phenolic acids | Phenolic acids | 149.06 | 106.04 | 150.07 | [M-H]- |
| 2,3-Dihydroxybenzoic Acid | Phenolic acids | Phenolic acids | 153.02 | 109.03 | 154.03 | [M-H]- |
| 2,4,5-Trimethoxybenzoic acid | Phenolic acids | Phenolic acids | 213.07 | 195.07 | 212.07 | [M+H]+ |
| 2,4,6-Trihydroxybenzoic acid | Phenolic acids | Phenolic acids | 171.03 | 81.03 | 170.02 | [M+H]+ |
| 2,4-Dihydroxybenzoic acid | Phenolic acids | Phenolic acids | 153.02 | 109.03 | 154.03 | [M-H]- |
| 2,5-Dihydroxybenzoic acid; Gentisic Acid | Phenolic acids | Phenolic acids | 153.02 | 109.03 | 154.03 | [M-H]- |
| 2,5-O-Di-CinnamylSorbitol | Phenolic acids | Phenolic acids | 415.2115 | 119.0861 | 414.20 | [M+H]+ |
| 2,6-Di-tert-butylphenol | Phenolic acids | Phenolic acids | 205.16 | 189.13 | 206.17 | [M-H]- |
| 2,6-Dimethoxybenzoic acid | Phenolic acids | Phenolic acids | 183.06 | 165.06 | 182.06 | [M+H]+ |
| 2,6-dimethoxybenzene-1,4-diol 1-O-β-D-glucopyranoside | Phenolic acids | Phenolic acids | 331.1 | 153.02 | 332.11 | [M-H]- |
| 2-(4-Hydroxycinnamoyl)glucoside | Phenolic acids | Phenolic acids | 531.15 | 205.05 | 532.16 | [M-H]- |
| 2-(Formylamino)benzoic acid | Phenolic acids | Phenolic acids | 164.04 | 120.05 | 165.04 | [M-H]- |
| 2-(Hydroxymethyl)Phenyl 2-O-Beta-D-Glucopyranosyl-Beta-D-Glucopyranoside | Phenolic acids | Phenolic acids | 507.17 | 179.06 | 448.16 | [M+CH3COOH-H]- |
| 2-Amino-3-methoxybenzoic acid | Phenolic acids | Phenolic acids | 168.06 | 94.06 | 167.06 | [M+H]+ |
| 2-Caffeoyl)rhamnosideO-rhamnoside | Phenolic acids | Phenolic acids | 471.15 | 323.08 | 472.16 | [M-H]- |
| 2-Feruloyl-6-(4-hydroxycinnamoyl)glucoside | Phenolic acids | Phenolic acids | 501.14 | 163.04 | 502.15 | [M-H]- |
| 2-Feruloyl-sn-glycerol | Phenolic acids | Phenolic acids | 269.1 | 177.05 | 268.09 | [M+H]+ |
| 2-Galloyl-6-O-Benzoyl Glucose | Phenolic acids | Phenolic acids | 435.09 | 169.01 | 436.10 | [M-H]- |
| 2-Hydroxy-3-(4-Hydroxyphenyl)Propanoic Acid | Phenolic acids | Phenolic acids | 181.05 | 135.04 | 182.06 | [M-H]- |
| 2-Hydroxy-3-phenylpropanoic acid | Phenolic acids | Phenolic acids | 165.06 | 103.06 | 166.06 | [M-H]- |
| 2-Hydroxycinnamic acid | Phenolic acids | Phenolic acids | 163.04 | 119.05 | 164.05 | [M-H]- |
| 2-Hydroxyphenylacetic acid | Phenolic acids | Phenolic acids | 151.04 | 107.05 | 152.05 | [M-H]- |
| 2-[4-(3-Hydroxypropyl)-2-methoxyphenoxy]-1,3-propanediol 1-glucoside | Phenolic acids | Phenolic acids | 477.1983 | 181.0866 | 418.18 | [M+CH3COO]- |
| 2-β-D-Glucopyranosyloxy-5-hydroxyphenylacetic acidmethylester | Phenolic acids | Phenolic acids | 343.1 | 181.05 | 344.11 | [M-H]- |
| 3'-O-Beta-D-Glucopyranosyl plumbagic acid | Phenolic acids | Phenolic acids | 385.11 | 179.07 | 386.12 | [M-H]- |
| 3,4'-Dihydroxy-3'-methoxybenzenepentanoic acid | Phenolic acids | Phenolic acids | 239.09 | 195.1 | 240.10 | [M-H]- |
| 3,4'-Dihydroxypropiophenone glucoside | Phenolic acids | Phenolic acids | 329.12 | 121.03 | 328.12 | [M+H]+ |
| 3,4,5-Trimethoxybenzoic acid methyl ester | Phenolic acids | Phenolic acids | 227.09 | 195.06 | 226.08 | [M+H]+ |
| 3,4,5-Trimethoxycinnamic acid | Phenolic acids | Phenolic acids | 237.08 | 103.02 | 238.08 | [M-H]- |
| 3,4-Dihydroxybenzoic acid (Protocatechuic acid) | Phenolic acids | Phenolic acids | 153.02 | 109.03 | 154.03 | [M-H]- |
| 3,4-Dimethoxybenzoic acid; Veratric acid | Phenolic acids | Phenolic acids | 183.06 | 165.05 | 182.06 | [M+H]+ |
| 3,4-Dimethoxycinnamic acid | Phenolic acids | Phenolic acids | 209.08 | 191.07 | 208.07 | [M+H]+ |
| 3,5-Dihydroxy-4-methoxybenzoic acid; 4-O-Methylgallic Acid | Phenolic acids | Phenolic acids | 183.03 | 139.04 | 184.04 | [M-H]- |
| 3,6-dimethoxyphenanthren-4-ol | Phenolic acids | Phenolic acids | 255.1 | 107.05 | 254.09 | [M+H]+ |
| 3-(3',4',5'-trimethoxyphenyl)-1,2-propanediol | Phenolic acids | Phenolic acids | 243.12 | 181.08 | 242.12 | [M+H]+ |
| 3-(4-Hydroxyphenyl)-propionic acid | Phenolic acids | Phenolic acids | 165.06 | 119.05 | 166.06 | [M-H]- |
| 3-(Hydroxycinnamoyl)-quinic acid | Phenolic acids | Phenolic acids | 337.09 | 163.04 | 338.10 | [M-H]- |
| 3-Aminosalicylic acid | Phenolic acids | Phenolic acids | 152.04 | 108.05 | 153.04 | [M-H]- |
| 3-Feruloyl-sucrose | Phenolic acids | Phenolic acids | 517.16 | 175.04 | 518.16 | [M-H]- |
| 3-Galloyl-6-O-Benzoyl Glucose | Phenolic acids | Phenolic acids | 435.09 | 169.02 | 436.10 | [M-H]- |
| 3-Hydroxy-4-methoxybenzoic acid; Isovanillic Acid | Phenolic acids | Phenolic acids | 169.05 | 65.04 | 168.04 | [M+H]+ |
| 3-Hydroxycinnamic Acid | Phenolic acids | Phenolic acids | 163.04 | 119.05 | 164.05 | [M-H]- |
| 3-Methoxybenzoic acid | Phenolic acids | Phenolic acids | 151.04 | 107.05 | 152.05 | [M-H]- |
| 3-Methylsalicylic Acid | Phenolic acids | Phenolic acids | 151.04 | 107.05 | 152.05 | [M-H]- |
| 3-O-(3,5-Dimethoxy-4-hydroxybenzoyl)-4-O-caffeoylquinic acid | Phenolic acids | Phenolic acids | 533.13 | 323.08 | 534.14 | [M-H]- |
| 3-O-Methylgallic acid | Phenolic acids | Phenolic acids | 183.03 | 124.02 | 184.04 | [M-H]- |
| 3-O-p-Coumaroyl Galactonic Acid | Phenolic acids | Phenolic acids | 341.09 | 195.05 | 342.10 | [M-H]- |
| 3-O-p-Coumaroylquinic acid | Phenolic acids | Phenolic acids | 337.09 | 191.06 | 338.10 | [M-H]- |
| 3-[(1-Carboxyvinyl)oxy]benzoic acid | Phenolic acids | Phenolic acids | 207.03 | 119.05 | 208.04 | [M-H]- |
| 3-[(2S,3S)-2-(4-hydroxy-3-methoxyphenyl)-7-methoxy-3-methyl-2,3-dihydro-1-benzofuran-5-yl]prop-2-enal | Phenolic acids | Phenolic acids | 339.12 | 309.08 | 340.13 | [M-H]- |
| 3-[4-(β-D-glucopyranoside)-phenylacrylic]-acid | Phenolic acids | Phenolic acids | 325.09 | 119.05 | 326.10 | [M-H]- |
| 3-hydroxy-5-methoxybenzaldehyde | Phenolic acids | Phenolic acids | 151.04 | 77.04 | 152.05 | [M-H]- |
| 4,7,9,9'-Tetrahydroxy-3,3'-dimethoxy-8-O-4'-neolignan | Phenolic acids | Phenolic acids | 377.1596 | 329.1389 | 378.17 | [M-H]- |
| 4-(3,4,5-Trihydroxybenzoxy)benzoic acid | Phenolic acids | Phenolic acids | 289.04 | 137.02 | 290.04 | [M-H]- |
| 4-(3-O-Sulfo-Beta-D-Glucopyranosyloxy)-3-Hydroxybenzoic Acid | Phenolic acids | Phenolic acids | 395.0276 | 153.0185 | 396.04 | [M-H]- |
| 4-(6-O-Sulfo-Beta-D-Glucopyranosyloxy)-3-Hydroxybenzoic Acid | Phenolic acids | Phenolic acids | 395.0277 | 153.0182 | 396.04 | [M-H]- |
| 4-Aminobenzoic acid | Phenolic acids | Phenolic acids | 138.06 | 120.04 | 137.05 | [M+H]+ |
| 4-Galloyl-6-O-Benzoyl Glucose | Phenolic acids | Phenolic acids | 435.09 | 169.01 | 436.10 | [M-H]- |
| 4-Hydroxybenzoic acid | Phenolic acids | Phenolic acids | 137.02 | 93.03 | 138.03 | [M-H]- |
| 4-Hydroxybenzoic acid glucosyl xyloside | Phenolic acids | Phenolic acids | 431.12 | 137.02 | 432.13 | [M-H]- |
| 4-Hydroxybenzoylmalic acid | Phenolic acids | Phenolic acids | 253.04 | 137.02 | 254.04 | [M-H]- |
| 4-Hydroxyphenyllactic Acid | Phenolic acids | Phenolic acids | 181.05 | 135.04 | 182.06 | [M-H]- |
| 4-Methoxycinnamic acid | Phenolic acids | Phenolic acids | 179.07 | 119.05 | 178.06 | [M+H]+ |
| 4-Methoxyphenylpropionic acid | Phenolic acids | Phenolic acids | 179.07 | 146.04 | 180.08 | [M-H]- |
| 4-O-(3'-O-alpha-D-Glucopyranosyl)caffeoylquinic acid | Phenolic acids | Phenolic acids | 515.14 | 191.06 | 516.15 | [M-H]- |
| 4-O-Glucosyl-4-hydroxybenzoic acid | Phenolic acids | Phenolic acids | 299.08 | 137.02 | 300.08 | [M-H]- |
| 4-O-Glucosyl-sinapate | Phenolic acids | Phenolic acids | 385.11 | 223.06 | 386.12 | [M-H]- |
| 5-Acetylsalicylic acid | Phenolic acids | Phenolic acids | 181.05 | 107.05 | 180.04 | [M+H]+ |
| 5-Glucosyloxy-2-Hydroxybenzoic acid methyl ester | Phenolic acids | Phenolic acids | 329.09 | 167.04 | 330.10 | [M-H]- |
| 5-O-p-Coumaroylquinic acid | Phenolic acids | Phenolic acids | 337.09 | 191.06 | 338.10 | [M-H]- |
| 5-hydroxy-1,7-bis(4-hydroxy-3-methoxyphenyl)hept-l-en-3-one | Phenolic acids | Phenolic acids | 373.17 | 177.06 | 372.16 | [M+H]+ |
| 6'-O-Feruloyl-D-sucrose | Phenolic acids | Phenolic acids | 517.16 | 175.04 | 518.16 | [M-H]- |
| 6-O-Caffeoyl-D-glucose | Phenolic acids | Phenolic acids | 341.09 | 179.03 | 342.10 | [M-H]- |
| 6-[4-(Acetyloxymethyl)phenoxy]-4-hydroxyoxane-2-carboxylic acid | Phenolic acids | Phenolic acids | 311.11 | 131.05 | 310.11 | [M+H]+ |
| Acropyrone | Phenolic acids | Phenolic acids | 225.08 | 119.05 | 224.07 | [M+H]+ |
| Anacardic acid | Phenolic acids | Phenolic acids | 349.27 | 123.08 | 348.27 | [M+H]+ |
| Anthranilate-1-O-Sophoroside | Phenolic acids | Phenolic acids | 460.15 | 117.9 | 461.15 | [M-H]- |
| Awsoniaside B | Phenolic acids | Phenolic acids | 371.13 | 209.08 | 372.14 | [M-H]- |
| Benzoic acid | Phenolic acids | Phenolic acids | 123.04 | 79.05 | 122.04 | [M+H]+ |
| Benzoylformic acid | Phenolic acids | Phenolic acids | 149.02 | 77.04 | 150.03 | [M-H]- |
| Benzoylmalic acid | Phenolic acids | Phenolic acids | 237.04 | 121.03 | 238.05 | [M-H]- |
| Benzoyltartaric acid | Phenolic acids | Phenolic acids | 253.04 | 121.03 | 254.04 | [M-H]- |
| Butyl isobutyl phthalate | Phenolic acids | Phenolic acids | 279.16 | 149.03 | 278.15 | [M+H]+ |
| Caffeic acid | Phenolic acids | Phenolic acids | 179.03 | 135.05 | 180.04 | [M-H]- |
| Caffeoylbenzoyltartaric acid | Phenolic acids | Phenolic acids | 415.07 | 179.03 | 416.07 | [M-H]- |
| Chicoric Acid | Phenolic acids | Phenolic acids | 473.07 | 149.01 | 474.08 | [M-H]- |
| Cimicifugic acid E-glucose | Phenolic acids | Phenolic acids | 595.17 | 287.06 | 594.16 | [M+H]+ |
| Cimicifugic acid E-glucose-rhamnose | Phenolic acids | Phenolic acids | 741.22 | 433.12 | 740.22 | [M+H]+ |
| Cinnamic acid | Phenolic acids | Phenolic acids | 147.05 | 103.06 | 148.05 | [M-H]- |
| DL-3-Phenyllactic acid | Phenolic acids | Phenolic acids | 165.06 | 119.05 | 166.06 | [M-H]- |
| Dibutyl phthalate | Phenolic acids | Phenolic acids | 279.16 | 149.02 | 278.15 | [M+H]+ |
| Dihydrocaffeic acid | Phenolic acids | Phenolic acids | 183.07 | 165.06 | 182.06 | [M+H]+ |
| Dihydrocaffeoylglucose | Phenolic acids | Phenolic acids | 343.1 | 181.05 | 344.11 | [M-H]- |
| Dihydroferulic Acid | Phenolic acids | Phenolic acids | 195.07 | 136.05 | 196.07 | [M-H]- |
| Dihydroferulic acid glucoside | Phenolic acids | Phenolic acids | 357.12 | 195.07 | 358.13 | [M-H]- |
| Diisobutyl phthalate | Phenolic acids | Phenolic acids | 279.16 | 149.02 | 278.15 | [M+H]+ |
| Erythro-Guaiacylglycerol | Phenolic acids | Phenolic acids | 213.08 | 150.03 | 214.08 | [M-H]- |
| Ethylparaben | Phenolic acids | Phenolic acids | 165.06 | 92.03 | 166.06 | [M-H]- |
| Ethylsalicylate | Phenolic acids | Phenolic acids | 167.07 | 121.03 | 166.06 | [M+H]+ |
| Eudesmic acid (3,4,5-trimethoxybenzoic acid) | Phenolic acids | Phenolic acids | 213.08 | 154.06 | 212.07 | [M+H]+ |
| Ferulic acid | Phenolic acids | Phenolic acids | 193.05 | 134.04 | 194.06 | [M-H]- |
| Furo(2,3-f)-1,3-bewnzodioxole | Phenolic acids | Phenolic acids | 271.06 | 149.02 | 270.05 | [M+H]+ |
| Gallic acid | Phenolic acids | Phenolic acids | 169.01 | 125.02 | 170.02 | [M-H]- |
| Gallic acid-1-O-xyloside | Phenolic acids | Phenolic acids | 301.06 | 168.01 | 302.06 | [M-H]- |
| Glucosyl tachioside | Phenolic acids | Phenolic acids | 463.14 | 121.03 | 464.15 | [M-H]- |
| Glucosyloxybenzoic acid | Phenolic acids | Phenolic acids | 299.0776 | 137.0239 | 300.08 | [M-H]- |
| Glucosyringic acid | Phenolic acids | Phenolic acids | 359.1 | 197.05 | 360.11 | [M-H]- |
| Grevilloside F | Phenolic acids | Phenolic acids | 341.09 | 135.04 | 342.10 | [M-H]- |
| Homogentisic acid | Phenolic acids | Phenolic acids | 167.03 | 123.05 | 168.04 | [M-H]- |
| Hydrocinnamic acid | Phenolic acids | Phenolic acids | 149.06 | 105.07 | 150.07 | [M-H]- |
| Hydroxyferulic acid glucoside | Phenolic acids | Phenolic acids | 371.1 | 209.05 | 372.11 | [M-H]- |
| Iriflophenone | Phenolic acids | Phenolic acids | 247.06 | 153.0184 | 246.05 | [M+H]+ |
| Isoamericanin A | Phenolic acids | Phenolic acids | 329.1 | 147.04 | 328.09 | [M+H]+ |
| Kelampayoside A[3,4,5-Trimethoxyphenol-β-D-apiosyl-(1→6)-β-D-glucoside] | Phenolic acids | Phenolic acids | 479.17 | 185.08 | 478.17 | [M+H]+ |
| Lavandulifolioside | Phenolic acids | Phenolic acids | 755.25 | 593.21 | 756.25 | [M-H]- |
| Mandelic acid | Phenolic acids | Phenolic acids | 151.04 | 107.05 | 152.05 | [M-H]- |
| Mandelic acid-β-glucoside | Phenolic acids | Phenolic acids | 313.09 | 101.02 | 314.10 | [M-H]- |
| Methyl (2Z)-3-(3,4-dihydroxyphenyl)-2-propenoate | Phenolic acids | Phenolic acids | 195.07 | 153.02 | 194.06 | [M+H]+ |
| Methyl 2,4-dihydroxyphenylacetate | Phenolic acids | Phenolic acids | 181.05 | 135.04 | 182.06 | [M-H]- |
| Methyl 3-(3-hydroxy-4-methoxyphenyl)propanoate | Phenolic acids | Phenolic acids | 209.08 | 179.03 | 210.09 | [M-H]- |
| Methyl 3-O-Beta-Glucopyranosyl-Gallate | Phenolic acids | Phenolic acids | 345.0829 | 183.0296 | 346.09 | [M-H]- |
| Methyl 3-O-glucosyl-4-hydroxybenzoate | Phenolic acids | Phenolic acids | 329.09 | 167.03 | 330.10 | [M-H]- |
| Methyl 4-hydroxybenzoate | Phenolic acids | Phenolic acids | 151.04 | 136.02 | 152.05 | [M-H]- |
| Methyl Hydroxycinnamate | Phenolic acids | Phenolic acids | 179.07 | 119.05 | 178.06 | [M+H]+ |
| Methyl Orsellinate | Phenolic acids | Phenolic acids | 183.06 | 123.04 | 182.06 | [M+H]+ |
| Methyl caffeate | Phenolic acids | Phenolic acids | 193.05 | 135.05 | 194.06 | [M-H]- |
| Methyl gallate | Phenolic acids | Phenolic acids | 183.03 | 124.02 | 184.04 | [M-H]- |
| Methyl-3-(3-hydroxyphenyl)Propionate | Phenolic acids | Phenolic acids | 181.09 | 121.06 | 180.08 | [M+H]+ |
| Methylgallic Acid 3-(6''-Sulfate)Glucoside | Phenolic acids | Phenolic acids | 425.0394 | 241.0028 | 426.05 | [M-H]- |
| Methylgalloyl-glucose-arabinoside | Phenolic acids | Phenolic acids | 477.13 | 183.02 | 478.13 | [M-H]- |
| Moracin Y | Phenolic acids | Phenolic acids | 271.06 | 153.05 | 270.05 | [M+H]+ |
| Mucic acid-1,4-lactone-2-O-gallate | Phenolic acids | Phenolic acids | 343.03 | 85.03 | 344.04 | [M-H]- |
| O-Anisic acid (2-Methoxybenzoic acid) | Phenolic acids | Phenolic acids | 151.04 | 93.03 | 152.05 | [M-H]- |
| Phenylpyruvic acid | Phenolic acids | Phenolic acids | 163.04 | 91.06 | 164.05 | [M-H]- |
| Phthalic acid | Phenolic acids | Phenolic acids | 165.02 | 77.04 | 166.03 | [M-H]- |
| Piperonylic acid | Phenolic acids | Phenolic acids | 165.02 | 91.02 | 166.03 | [M-H]- |
| Proglobeflowery acid | Phenolic acids | Phenolic acids | 235.1 | 220.07 | 236.10 | [M-H]- |
| Protocatechuic Acid Methyl Ester | Phenolic acids | Phenolic acids | 167.04 | 108.02 | 168.04 | [M-H]- |
| Protocatechuic acid 1-O-(Glucosylvanilloyl) | Phenolic acids | Phenolic acids | 465.1 | 303.05 | 466.11 | [M-H]- |
| Protocatechuic acid 1-O-Rutinoside | Phenolic acids | Phenolic acids | 461.13 | 152.01 | 462.14 | [M-H]- |
| Protocatechuic acid glucosyl xyloside | Phenolic acids | Phenolic acids | 447.12 | 152.01 | 448.12 | [M-H]- |
| Protocatechuic acid-4-O-glucoside | Phenolic acids | Phenolic acids | 315.07 | 153.02 | 316.08 | [M-H]- |
| Pyrafortunoside A | Phenolic acids | Phenolic acids | 475.14 | 167.03 | 476.15 | [M-H]- |
| Regaloside G | Phenolic acids | Phenolic acids | 429.14 | 193.05 | 430.15 | [M-H]- |
| Rhamnosyl-gentisic acid-5-O-β-D-glucoside | Phenolic acids | Phenolic acids | 461.13 | 153.02 | 462.14 | [M-H]- |
| Rosmarinic acid-3'-O-glucoside | Phenolic acids | Phenolic acids | 521.13 | 359.08 | 522.14 | [M-H]- |
| Salicylic acid-2-O-glucoside | Phenolic acids | Phenolic acids | 299.08 | 137.02 | 300.08 | [M-H]- |
| Salidroside | Phenolic acids | Phenolic acids | 299.1115 | 137.0245 | 300.12 | [M-H]- |
| Salvianic acid B | Phenolic acids | Phenolic acids | 359.08 | 197.05 | 360.09 | [M-H]- |
| Sinapic acid | Phenolic acids | Phenolic acids | 223.06 | 193.02 | 224.07 | [M-H]- |
| Sinapinaldehyde | Phenolic acids | Phenolic acids | 207.07 | 192.04 | 208.07 | [M-H]- |
| Syringic acid | Phenolic acids | Phenolic acids | 197.05 | 123.01 | 198.05 | [M-H]- |
| Syringic acid 4-O-rhamnoside | Phenolic acids | Phenolic acids | 343.1 | 197.05 | 344.11 | [M-H]- |
| Terephthalic acid | Phenolic acids | Phenolic acids | 165.0193 | 121.0295 | 166.03 | [M-H]- |
| Trans-5-O-(p-Coumaroyl)shikimate | Phenolic acids | Phenolic acids | 321.1 | 147.04 | 320.09 | [M+H]+ |
| Tropic acid | Phenolic acids | Phenolic acids | 165.06 | 103.06 | 166.06 | [M-H]- |
| Vanillic acid | Phenolic acids | Phenolic acids | 167.03 | 108.02 | 168.04 | [M-H]- |
| Veratraldehyde | Phenolic acids | Phenolic acids | 167.07 | 124.05 | 166.06 | [M+H]+ |
| galloyl xylosyl glucoside | Phenolic acids | Phenolic acids | 463.11 | 168.01 | 464.12 | [M-H]- |
| mudanoside B | Phenolic acids | Phenolic acids | 463.11 | 168.01 | 464.12 | [M-H]- |
| p-Coumaric acid | Phenolic acids | Phenolic acids | 165.05 | 119.05 | 164.05 | [M+H]+ |
| p-Coumaric acid ethyl ester | Phenolic acids | Phenolic acids | 193.09 | 147.04 | 192.08 | [M+H]+ |
| p-Coumaric acid methyl ester | Phenolic acids | Phenolic acids | 179.07 | 147.04 | 178.06 | [M+H]+ |
| p-Coumaric acid-4-O-glucoside | Phenolic acids | Phenolic acids | 325.09 | 163.04 | 326.10 | [M-H]- |
| p-Coumaroylmalic acid | Phenolic acids | Phenolic acids | 279.05 | 163.04 | 280.06 | [M-H]- |
| plantalloside | Phenolic acids | Phenolic acids | 639.2 | 639.19 | 640.20 | [M-H]- |
| α-Hydroxycinnamic Acid | Phenolic acids | Phenolic acids | 163.04 | 119.05 | 164.05 | [M-H]- |
| Arecatannin B1 | Tannins | Proanthocyanidins | 867.21 | 715.2 | 866.21 | [M+H]+ |
| Arecatannin C1 | Tannins | Proanthocyanidins | 867.21 | 715.2 | 866.21 | [M+H]+ |
| Procyanidin B1 | Tannins | Proanthocyanidins | 577.14 | 425.09 | 578.14 | [M-H]- |
| Procyanidin B2 | Tannins | Proanthocyanidins | 577.14 | 407.08 | 578.14 | [M-H]- |
| Procyanidin B3 | Tannins | Proanthocyanidins | 577.14 | 407.08 | 578.14 | [M-H]- |
| Procyanidin C2 | Tannins | Proanthocyanidins | 865.2 | 713.16 | 866.21 | [M-H]- |
| Theaflavin | Tannins | Proanthocyanidins | 565.13 | 427.1 | 564.13 | [M+H]+ |
| 3,3',4-O-Trimethylellagic acid | Tannins | Tannin | 345.06 | 313.03 | 344.05 | [M+H]+ |
| 3,3'-O-Dimethylellagic Acid | Tannins | Tannin | 329.03 | 298.98 | 330.04 | [M-H]- |
| 3-O-Methylellagic acid | Tannins | Tannin | 315.01 | 299.99 | 316.02 | [M-H]- |
| Ellagic acid-4-O-Xyloside | Tannins | Tannin | 433.04 | 301 | 434.05 | [M-H]- |
| Ellagic acid-4-O-glucoside | Tannins | Tannin | 463.05 | 300.99 | 464.06 | [M-H]- |
| Epiafzelechin-(4b->6)-Epicatechin 3,3'-Digallate | Tannins | Tannin | 865.1644 | 713.1519 | 866.17 | [M-H]- |
| Petasiphenol | Tannins | Tannin | 343.0818 | 179.0347 | 344.09 | [M-H]- |
| Theaflagallin | Tannins | Tannin | 399.07 | 233.05 | 400.08 | [M-H]- |

**Table S2 Antioxidant capacity of CO in different TPC groups**

|  | FRAP | DPPH | ABTS |
| --- | --- | --- | --- |
| CML | 4.40 ± 2.70a | 5.60 ± 3.87a | 19.15 ± 4.67b |
| CMM | 3.26 ± 1.60a | 6.71 ± 4.29a | 19.18 ± 4.19b |
| CMH | 5.56 ± 3.42a | 11.52 ± 11.97a | 29.29 ± 6.54a |

**Table S3 Information on 21 common differential phenolic markers**

| Index | Compounds | Class I | Class II | FRH/FRL | DPH/DPL | ABH/ABL |
| --- | --- | --- | --- | --- | --- | --- |
| M122 | 3-Hydroxy-4-methoxybenzoic acid; Isovanillic Acid | Phenolic acids | Phenolic acids | 1.37 | 1.46 | 1.30 |
| M750 | ulopterol | Lignans and Coumarins | Coumarins | 1.47 | 1.88 | 1.35 |
| M74 | 2-Hydroxyphenylacetic acid | Phenolic acids | Phenolic acids | 1.45 | 1.66 | 1.37 |
| M517 | Mandelic acid | Phenolic acids | Phenolic acids | 2.08 | 2.19 | 1.49 |
| M291 | Cirsilineol | Flavonoids | Flavanones | 1.61 | 1.31 | 1.54 |
| M126 | 3-Methylsalicylic Acid | Phenolic acids | Phenolic acids | 1.83 | 1.81 | 1.55 |
| M712 | Wogonin | Flavonoids | Flavones | 1.85 | 2.24 | 1.55 |
| M44 | 2',4,4'-Trihydroxychalcone | Flavonoids | Chalcones | 1.85 | 2.26 | 1.65 |
| M124 | 3-Methoxybenzoic acid | Phenolic acids | Phenolic acids | 1.85 | 1.86 | 1.69 |
| M136 | 3-hydroxy-5-methoxybenzaldehyde | Phenolic acids | Phenolic acids | 2.44 | 2.09 | 1.75 |
| M404 | Hydroxy coumestrol | Lignans and Coumarins | Coumarins | 2.16 | 2.47 | 1.88 |
| M51 | 2,4,2',4'-tetrahydroxy-3'-prenylchalcone | Flavonoids | Chalcones | 1.82 | 1.99 | 1.96 |
| M355 | Erythro-Guaiacylglycerol-β-Coniferyl Ether | Lignans and Coumarins | Lignans | 2.61 | 2.54 | 2.09 |
| M325 | Dimethylfraxetin; 6,7,8-Trimethoxycoumarin | Lignans and Coumarins | Coumarins | 2.81 | 2.86 | 2.26 |
| M169 | 5,6,7-Trimethoxycoumarin | Lignans and Coumarins | Coumarins | 2.61 | 2.78 | 2.28 |
| M196 | 6-[4-(Acetyloxymethyl)phenoxy]-4-hydroxyoxane-2-carboxylic acid | Phenolic acids | Phenolic acids | 2.43 | 2.11 | 2.55 |
| M52 | 2,4,4'-trihydroxychalcone | Flavonoids | Chalcones | 2.52 | 2.84 | 2.65 |
| M450 | Justicidin C | Lignans and Coumarins | Lignans | 2.66 | 3.09 | 3.31 |
| M674 | Skullcapflavone II | Flavonoids | Flavones | 4.03 | 2.01 | 4.47 |
| M536 | Methyl Orsellinate | Phenolic acids | Phenolic acids | 10.02 | 10.58 | 8.74 |
| M42 | 2',3',4',5,7-pentahydroxyflavone | Flavonoids | Flavones | 94.68 | 125.91 | 75.95 |

**Table S4** Literature reports related to camellia phenolics

| Research Topic | | Antioxidant | | Pretreatments | | Species | Extraction methods |
| --- | --- | --- | --- | --- | --- | --- | --- |
| Flavonoids | Flavones | 116 | 3 | 22 | 10% | 2 | 1 |
|  | Flavanols | 16 | 4 |  |  | 3 |  |
|  | Flavonols | 110 | 3 | 33 |  | 3 | 7 |
|  | Flavanones | 46 | 1 | 11 | 9% |  |  |
|  | Isoflavones | 30 |  |  | 10% |  |  |
|  | Other Flavonoids | 27 |  |  |  |  |  |
|  | Chalcones | 26 |  |  | 1% |  |  |
|  | Dihydrochalcones |  |  |  |  |  |  |
|  | Anthocyanidins | 15 |  |  | 3% |  |  |
|  | Flavanonols | 6 | 1 |  |  |  |  |
|  | Aurones | 3 |  |  |  |  |  |
|  | Isoflavonoids |  |  |  | 3% |  |  |
|  | Dihydroflavones |  |  |  |  | 2 | 1 |
|  | Dihydroisoflavones | 1 |  |  |  |  |  |
|  | flavan-3-ols |  |  | 12 |  |  |  |
| Lignans and Coumarins | Lignans | 115 |  |  | 5% |  |  |
|  | Coumarins | 37 |  |  | 3% |  |  |
| Phenolic acids | Phenolic acids | 188 | 12 | 76 |  | 13 | 6 |
| Tannins | Tannin | 8 |  |  |  |  |  |
|  | Proanthocyanidins | 7 |  |  |  |  |  |
| Others | stilbenes |  |  | 5 |  |  |  |
|  | Others |  | 1 | 3 | 56% |  | 2 |
| Total | | 751 |  | 162 | 105 | 23 | 17 |
| Reference | | This study | (Wang et al., 2021) | (Zheng et al., 2022) | (Wang et al., 2022) | (Wang et al., 2017) | (Lu et al., 2023) |

**Reference**

Lu, Y., Hou, R., Li, M., Yu, N., Huan, W., Nie, X., & Meng, X. (2023). Assessments of extraction methods onto the phenolic profiles and antioxidant properties in Camellia oleifera Abel oils. *European Food Research and Technology, 249*(7), 1875-1885. <https://doi.org/10.1007/s00217-023-04261-6>.

Wang, M., Zhang, Y., Wan, Y., Zou, Q., Shen, L., Fu, G., & Gong, E. S. (2022). Effect of pretreatments of camellia seeds on the quality, phenolic profile, and antioxidant capacity of camellia oil. *Front Nutr, 9*, 1023711. <https://doi.org/10.3389/fnut.2022.1023711>.

Wang, X., Contreras, M. d. M., Xu, D., Jia, W., Wang, L., & Yang, D. (2021). New insights into free and bound phenolic compounds as antioxidant cluster in tea seed oil: Distribution and contribution. *LWT, 136*. <https://doi.org/10.1016/j.lwt.2020.110315>.

Wang, X., Zeng, Q., Del Mar Contreras, M., & Wang, L. (2017). Profiling and quantification of phenolic compounds in Camellia seed oils: Natural tea polyphenols in vegetable oil. *Food Res Int, 102*, 184-194. <https://doi.org/10.1016/j.foodres.2017.09.089>.

Wei, Z., Yang, K., Guo, M., Luan, X., Duan, Z., & Li, X. (2022). The effect of thermal pretreatment processing on the distribution of free and bound phenolics in virgin Camellia oleifera seed oil. *LWT, 161*. <https://doi.org/10.1016/j.lwt.2022.113349>.

(B)

(A)


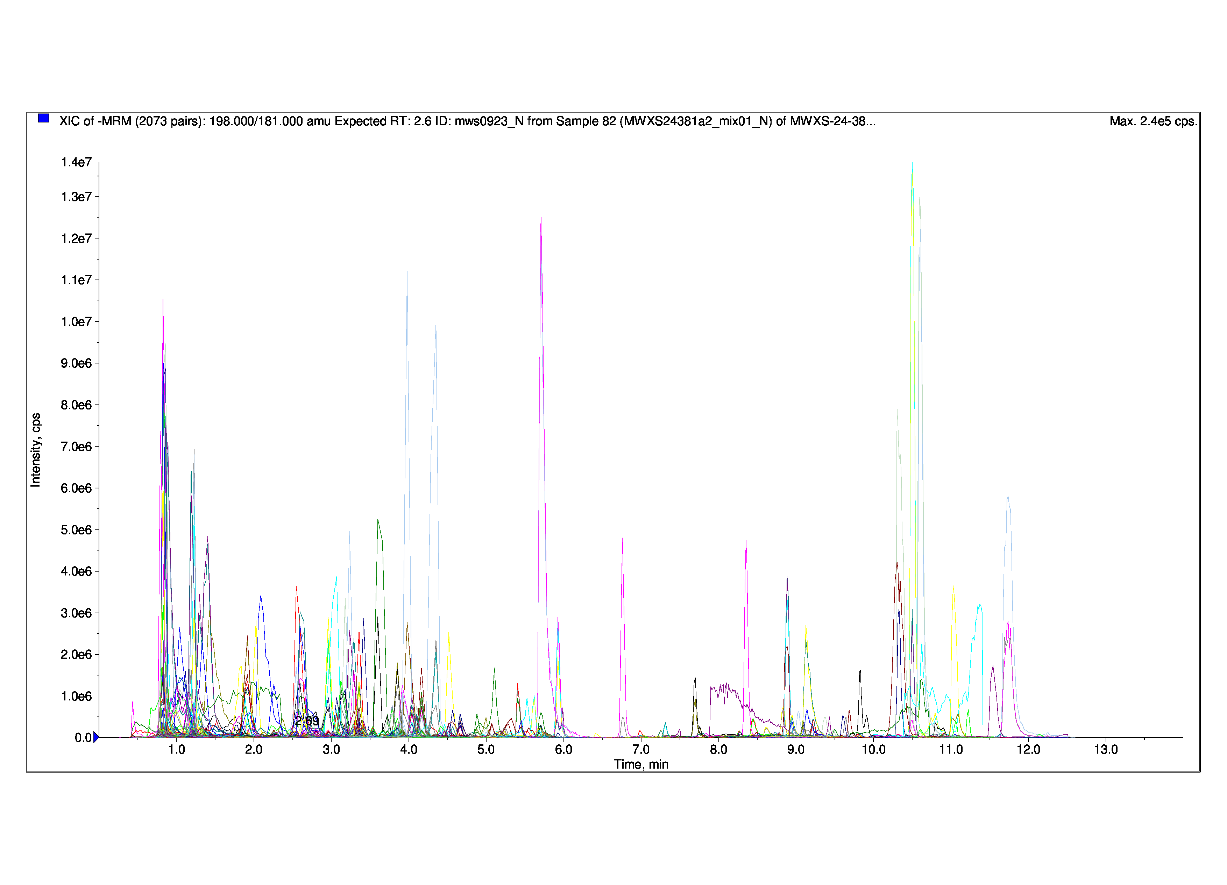

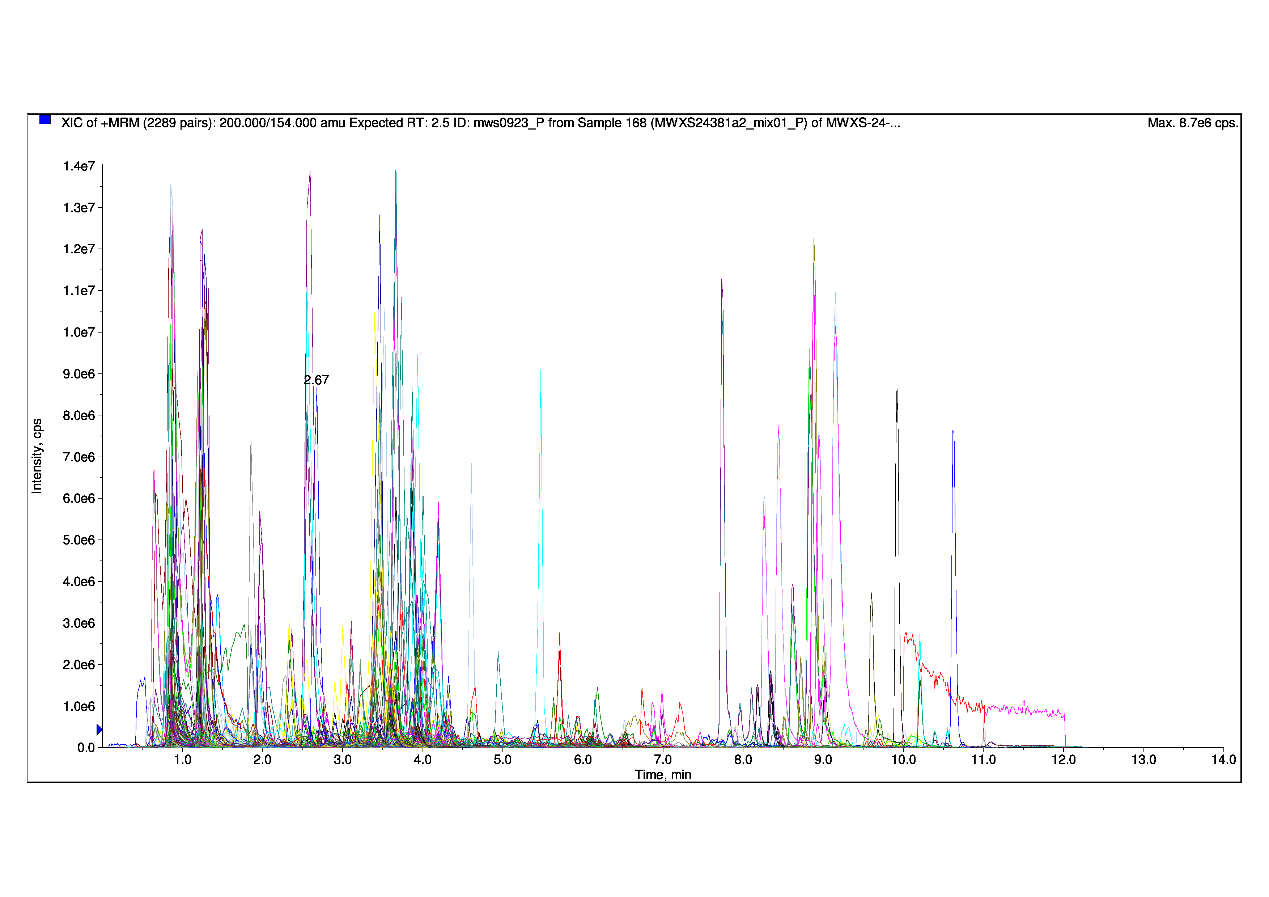


**Figure S1** Multiple peak plot of MRM metabolite assay. (A) Negative Ion Mode. (B) Positive Ion Mode.


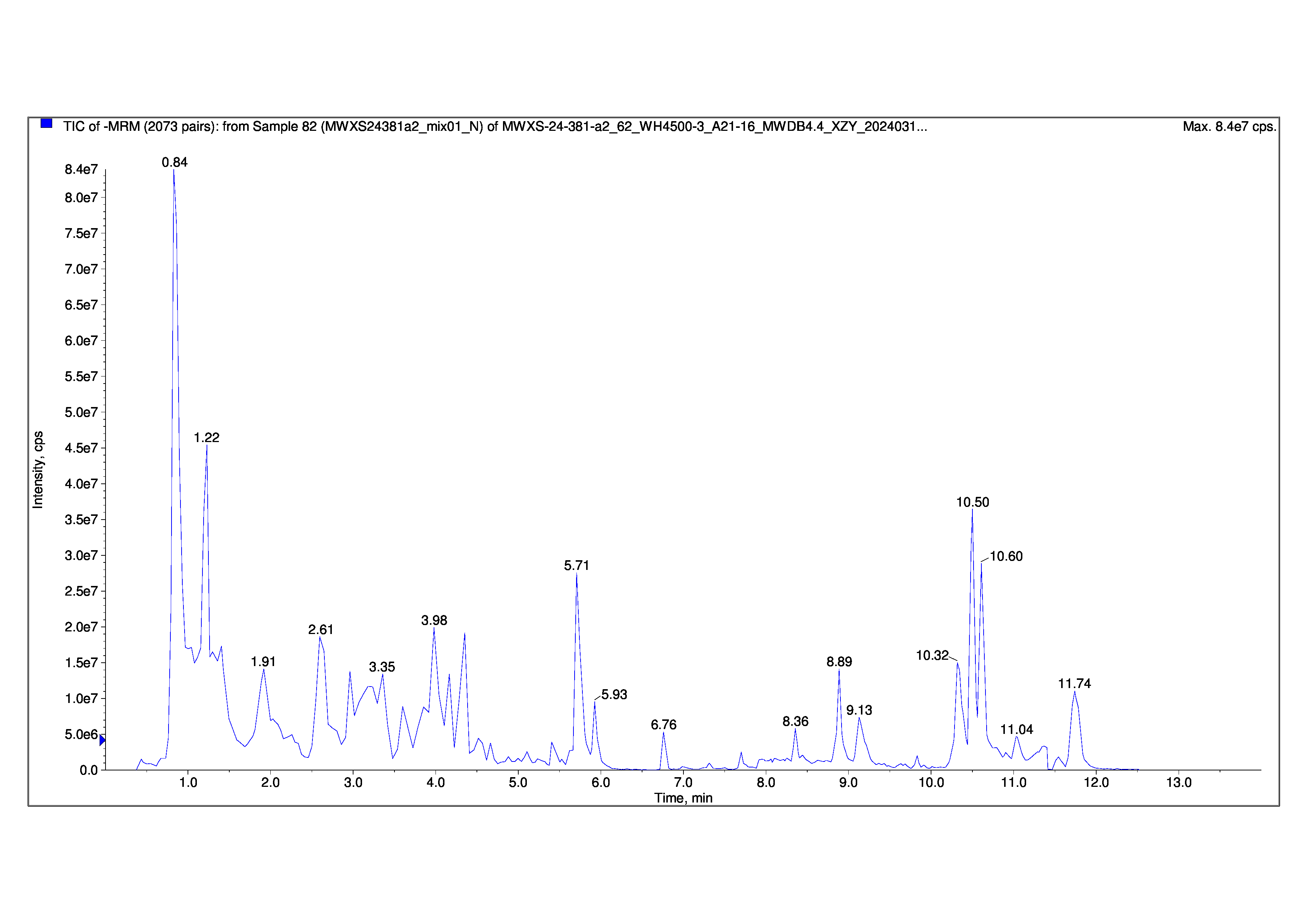

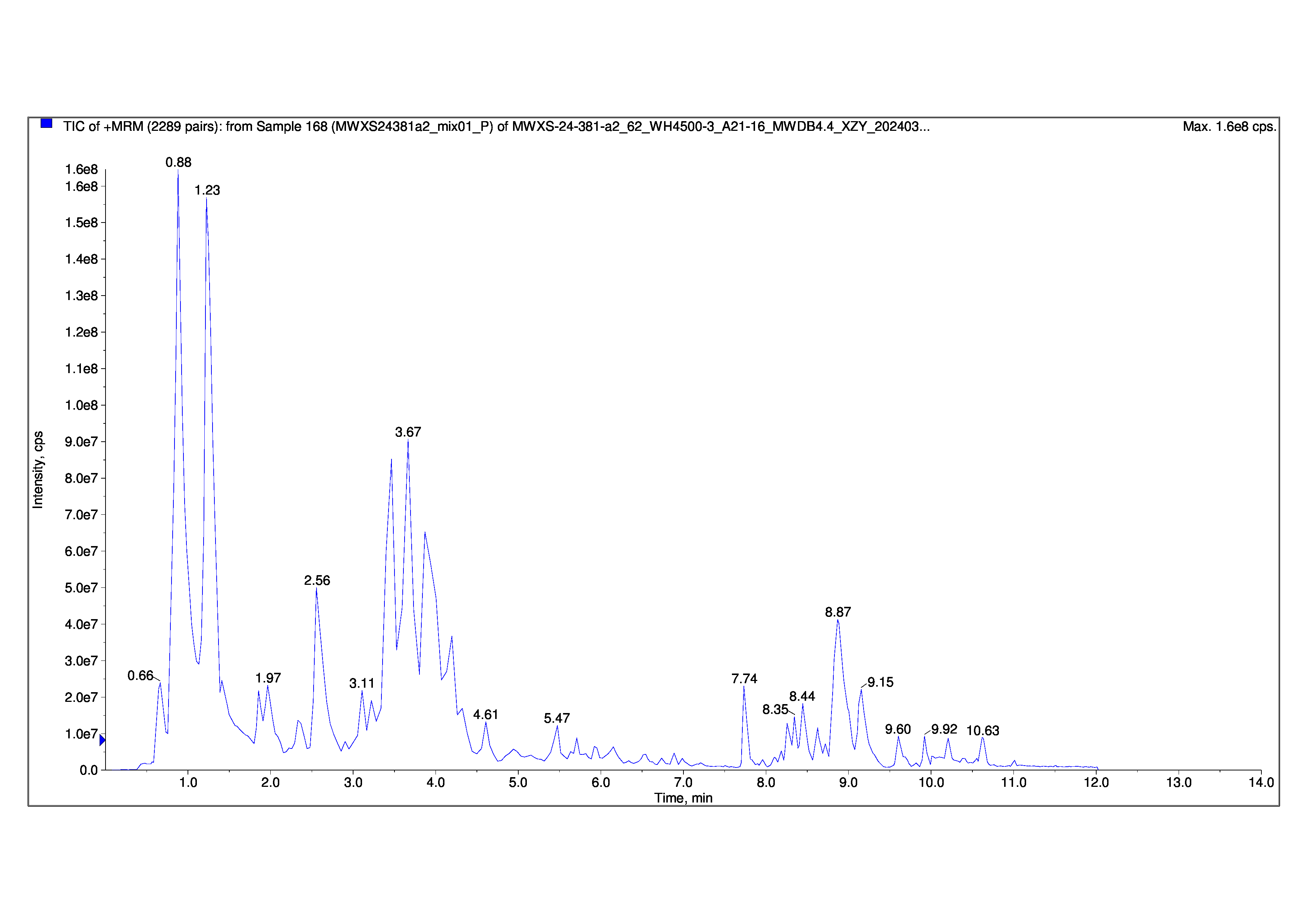


(B)

(A)

**Figure S2** Mixed-sample mass spectrometry analysis of total ion flow plots. (A) Negative Ion Mode. (B) Positive Ion Mode.


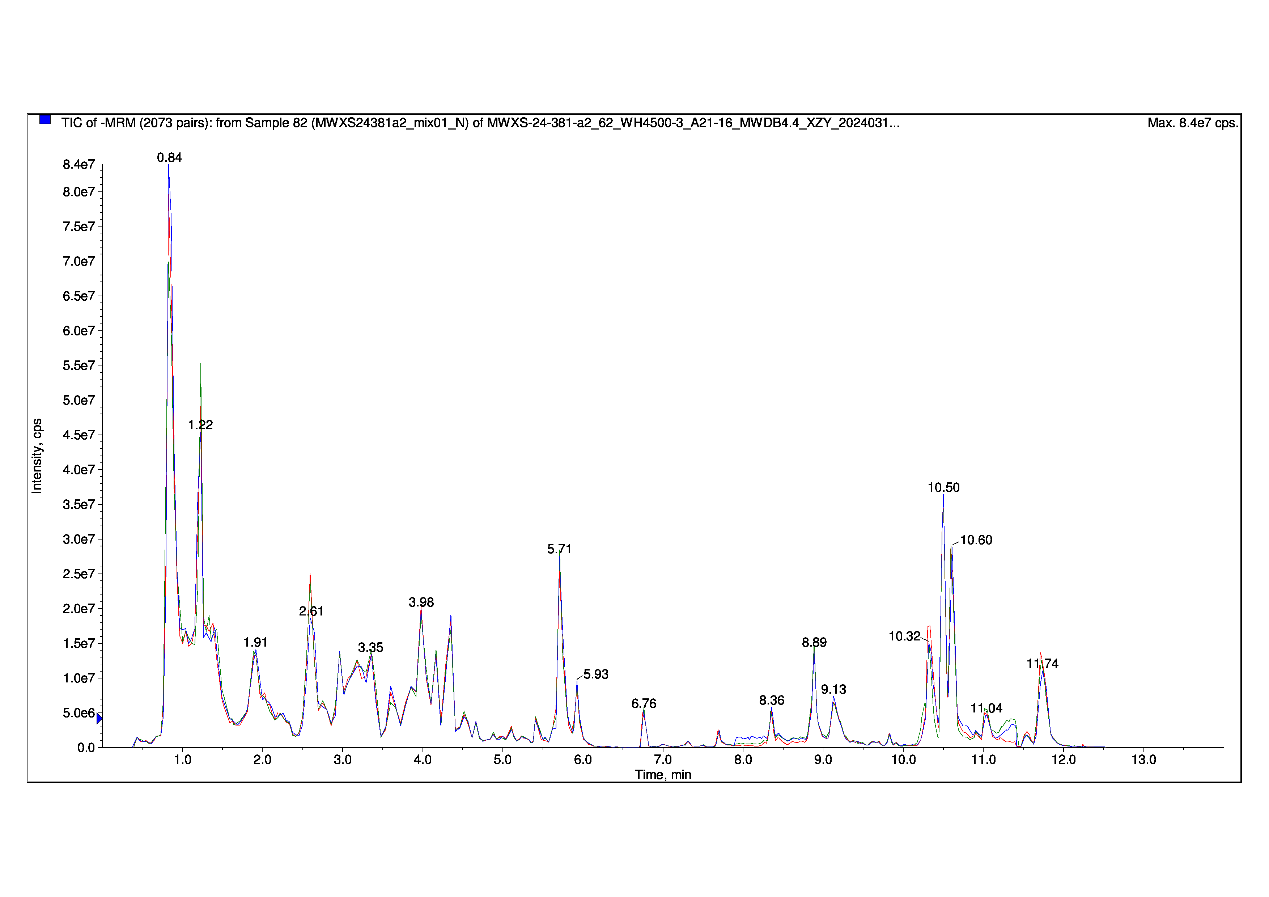

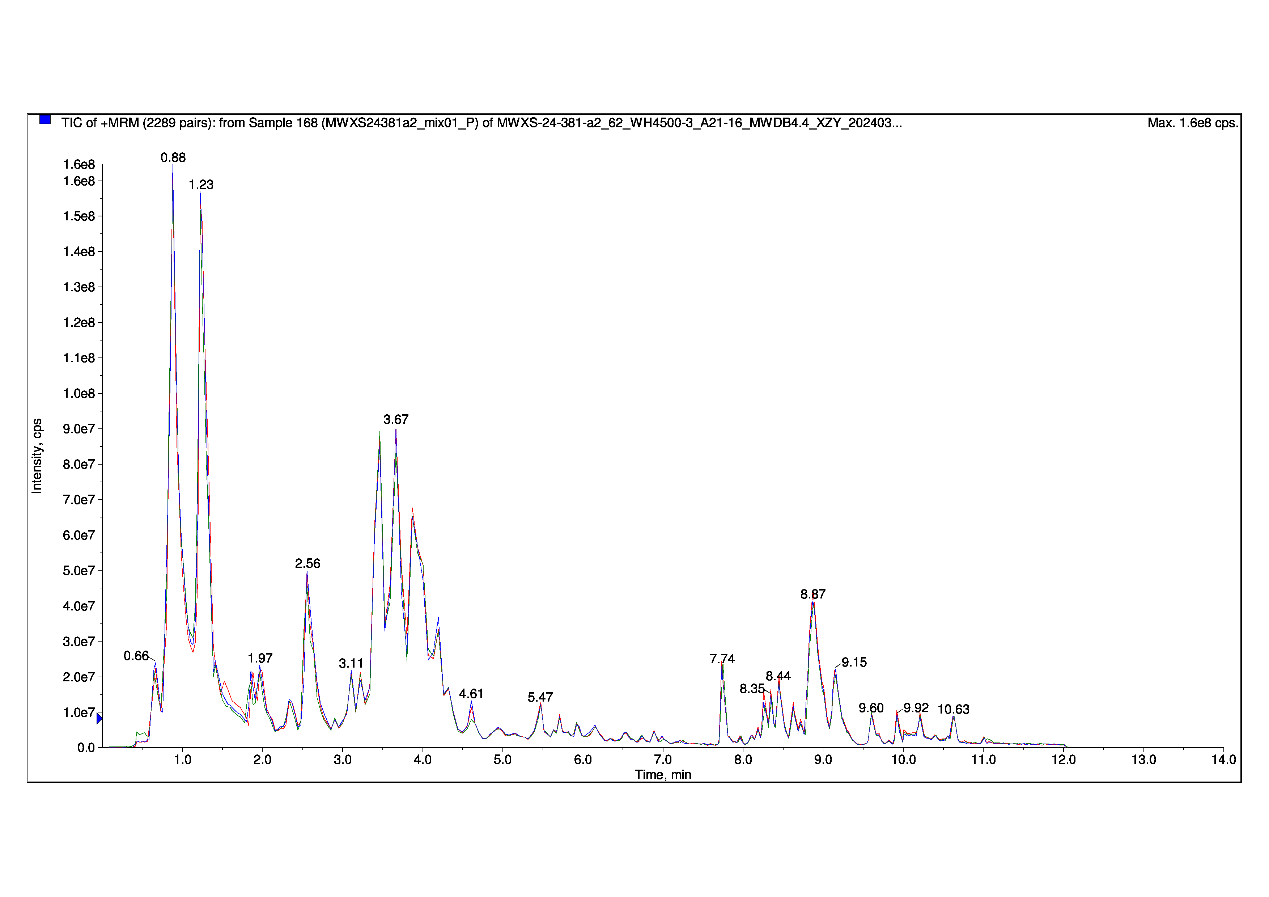


(A)

(B)

**Figure S3** Overlay of TIC for mass spectrometry detection of QC samples. (A) Negative Ion Mode. (B) Positive Ion Mode

(B)

(A)


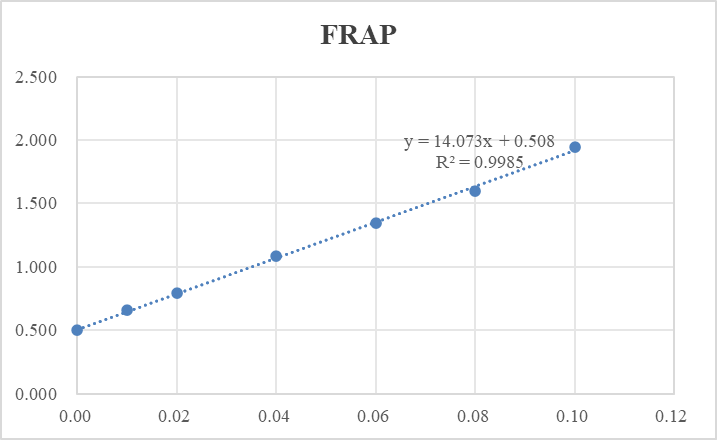

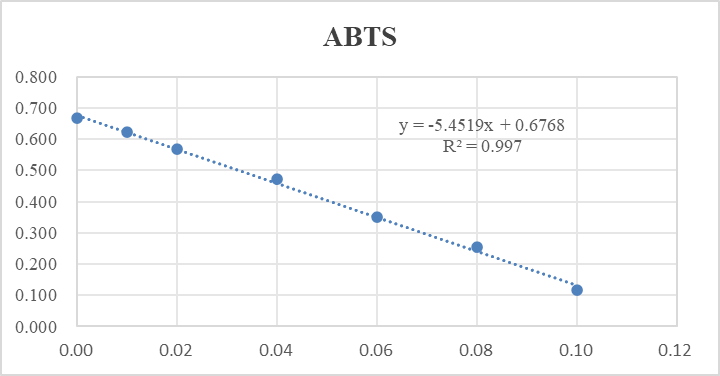

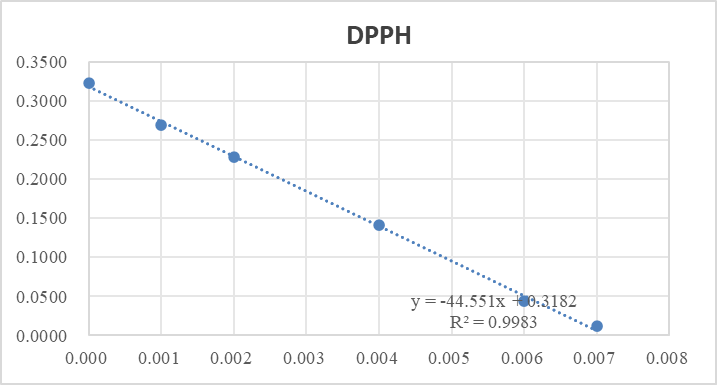


(C)

**Figure S4** The linear regression equation for the standard calibration curves of vitamin C. (A) FRAP. (B) DPPH. (C) ABTS.


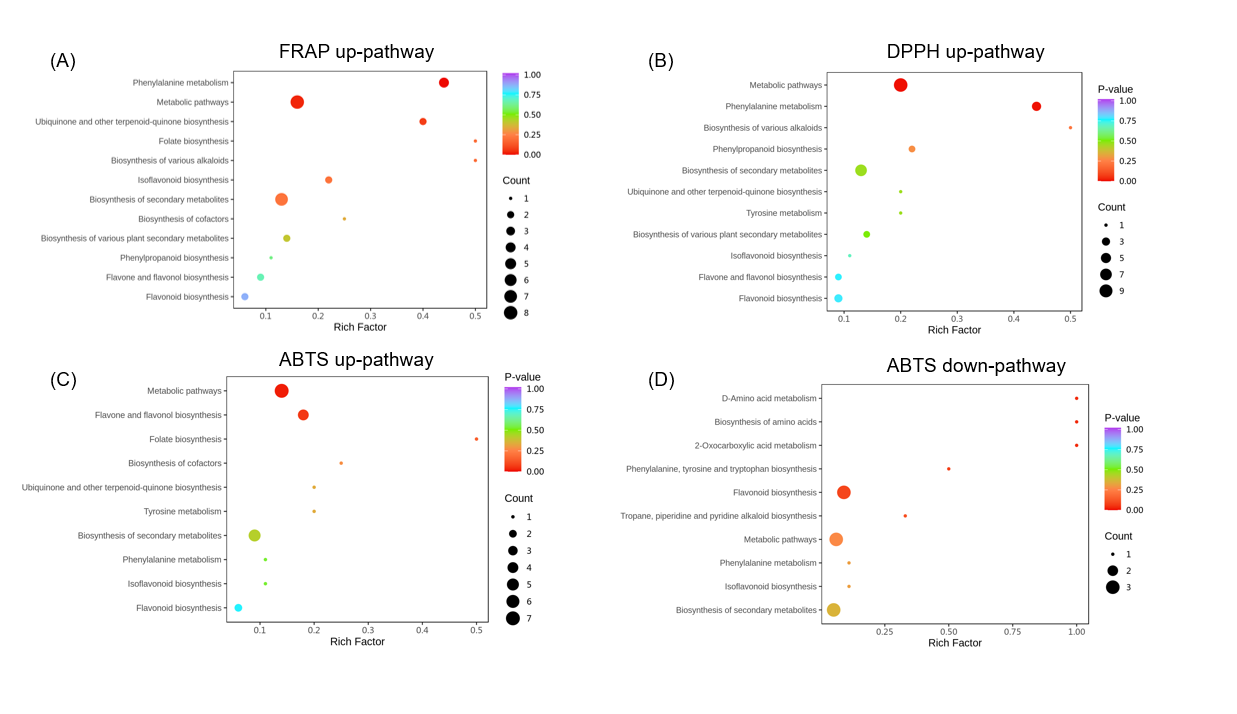


**Figure S5.** (A) Diagram of up-regulated metabolic pathways reflecting differences in FRL and FRH. (B) Diagram of up-regulated metabolic pathways reflecting differences in DPL and DPH. (C) Diagram of up-regulated metabolic pathways reflecting differences in ABL and ABH. (D) Diagram of down-regulated metabolic pathways reflecting differences in ABL and ABH.
